# Greater wealth inequality, less polygyny: rethinking the polygyny threshold model

**DOI:** 10.1098/rsif.2018.0035

**Published:** 2018-07-18

**Authors:** Cody T. Ross, Monique Borgerhoff Mulder, Seung-Yun Oh, Samuel Bowles, Bret Beheim, John Bunce, Mark Caudell, Gregory Clark, Heidi Colleran, Carmen Cortez, Patricia Draper, Russell D. Greaves, Michael Gurven, Thomas Headland, Janet Headland, Kim Hill, Barry Hewlett, Hillard S. Kaplan, Jeremy Koster, Karen Kramer, Frank Marlowe, Richard McElreath, David Nolin, Marsha Quinlan, Robert Quinlan, Caissa Revilla-Minaya, Brooke Scelza, Ryan Schacht, Mary Shenk, Ray Uehara, Eckart Voland, Kai Willführ, Bruce Winterhalder, John Ziker

**Affiliations:** 1 Behavioral Sciences Program, Santa Fe Institute, Santa Fe, NM, USA; 2 Department of Human Behavior, Ecology and Culture, Max Planck Institute for Evolutionary Anthropology, Leipzig, Germany; 3 Department of Anthropology, University of California, Davis, CA, USA; 4 Korea Insurance Research Institute, Yeongdeungpo-gu, Republic of Korea; 5 Allen School for Global Animal Health, Washington State University, Pullman, WA, USA; 6 Dept. of Linguistic and Cultural Evolution, Max Planck Institute for the Science of Human History, Leipzig, Germany; 7 Department of Anthropology, University of Nebraska, Lincoln, NE, USA; 8 Department of Anthropology, University of Utah, Logan, UT, USA; 9 Department of Anthropology, University of California, Santa Barbara, CA, USA; 10 SIL International, Dallas, TX, USA; 11 School of Human Evolution and Social Change, Arizona State University, Tempe, AZ, USA; 12 Department of Anthropology, Washington State University, Vancouver, WA, USA; 13 Department of Anthropology, University of New Mexico, Albuquerque, NM, USA; 14 Department of Anthropology, University of Cincinnati, Cincinnati, OH, USA; 15 Department of Biological Anthropology, University of Cambridge, UK; 16 Department of Anthropology, Pennsylvania State University, Philadelphia, PA, USA; 17 Department of Anthropology, Vanderbilt University, Nashville, TN, USA; 18 Department of Anthropology, University of California, Los Angeles, CA, USA; 19 Institut für Philosophie der Universität Giessen, Giessen, Germany; 20 Department of Anthropology, Boise State University, Boise, ID, USA; 21 Carl von Ossietzky University of Oldenburg, Oldenburg, Germany

**Keywords:** polygyny, monogamy, marriage systems, wealth inequality, behavioural ecology, evolutionary anthropology

## Abstract

Monogamy appears to have become the predominant human mating system with the emergence of highly unequal agricultural populations that replaced relatively egalitarian horticultural populations, challenging the conventional idea—based on the polygyny threshold model—that polygyny should be positively associated with wealth inequality. To address this polygyny paradox, we generalize the standard polygyny threshold model to a mutual mate choice model predicting the fraction of women married polygynously. We then demonstrate two conditions that are jointly sufficient to make monogamy the predominant marriage form, even in highly unequal societies. We assess if these conditions are satisfied using individual-level data from 29 human populations. Our analysis shows that with the shift to stratified agricultural economies: (i) the population frequency of relatively poor individuals increased, increasing wealth inequality, but decreasing the frequency of individuals with sufficient wealth to secure polygynous marriage, and (ii) diminishing marginal fitness returns to additional wives prevent extremely wealthy men from obtaining as many wives as their relative wealth would otherwise predict. These conditions jointly lead to a high population-level frequency of monogamy.

## 1. Introduction

Decades of both theoretical [1–4] and empirical research [5–9] based on the polygyny threshold model [1,2] have suggested that polygyny should be more common and more pronounced in populations in which males differ substantially in resource control. In humans, this will be in socio-cultural contexts where wealth is held predominately by men, and where there is high inequality in its distribution. Historical and cross-cultural records, however, suggest that polygyny became less common as relatively egalitarian horticultural production systems (land-abundant, labour-limited cultivation) transitioned into agricultural production systems (land-limited cultivation, often coupled with stratified social systems) [10,11], in spite of the fact that agriculture is characterized by both a greater importance of material wealth in the production process and greater levels of material wealth inequality than horticulture [12–15]. This is the polygyny paradox.

Existing hypotheses for the rise of monogamy with historic agricultural populations invoke the increasing importance of rival^1^ material wealth among agriculturalists [17], inheritance rules in conjunction with paternity certainty [4], male power relations [18], declines in female contributions to production [19,20], pathogen risk and punishment [21,22] and cultural group selection via the imposition of norms [23,24]. Since human behavioural variation is often determined by many underlying factors, there are likely to be complementary effects among the potential causes identified in these hypotheses.

Specifically, there should be coevolutionary interactions between the individual-level, economic- and fitness-based explanations for the rise of monogamy advanced here, and the cultural evolutionary explanations provided by Henrich *et al.* [24] and Bauch & McElreath [22]. Our results show how individual fitness maximization can explain the *de novo* origins of predominant monogamy within highly unequal populations. Should monogamy have group-level fitness benefits as suggested by Henrich *et al.* [24], its emergence in specific groups via the mechanism we propose would provide the source populations for cultural group selection [25] dynamics to propagate monogamy to other populations.

Explanations for the rise of monogamy in agricultural societies in the spirit of Alexander [23] and Henrich *et al.* [24] develop the idea that powerful leaders might have imposed monogamy on the masses because such a marriage norm leads to greater in-group male–male cooperation, improving the success of the group in inter-group contests (including warfare) [26]. The economically grounded explanation for the rise of monogamy that we present here is not necessarily in competition with such theories. Our model, however, establishes that basic changes in the structuring of wealth inequality coinciding with the rise of class-based societies would have made monogamy adaptive at the individual level in a large fraction of the population—greatly increasing the scope for hypotheses advancing hierarchical imposition or even frequency dependent social transmission of norms for monogamy.

The present analysis builds on work recognizing the importance of inherited wealth in structuring family relationships [4,17,27]. To this existing literature, we introduce a new individual-level, cross-cultural dataset of wealth, marriage and reproductive outcomes, numbering 11 813 records from 29 human populations, including hunter–gathers, horticulturalists, agropastoralists and agriculturalists. Our dataset is unusual in both its scope and in the availability of individual-level information, rather than qualitative societal summaries. While not without its limitations—discussed in more detail throughout—it captures the core features of the polygyny paradox.

Following Oh *et al.* [17], we develop a model of the equilibrium fraction of women married polygynously in a population where the extent of polygyny is determined by the fitness maximizing choices of both men and women. In contrast to the standard polygyny threshold model [1,2], which is a one-sided mate choice model that allows only for female choice, we develop a mutual or two-sided model [28]. In this model, male choice refers not to selecting particular females on the basis of their quality (females are identical in our model), but rather to the male's choice of the number of wives that will maximize his fitness. A male's demand for wives depends on his level of wealth and the costs of mating investment, and can be more than, less than or equal to the total number of women who would choose to marry him. Mutual mate choice is rare in nature, but the conditions for it are met in species in which biparental care is important for the survival of offspring, as is typically true of humans [29].

From our theoretical model, we identify two conditions that jointly can lead to a decrease in the population-level frequency of polygyny in highly unequal agricultural populations: (i) in these highly stratified economies, the fraction of men with sufficient wealth to make polygynous marriage an attractive option for them and their potential partners is low relative to other subsistence systems, and (ii) decreasing marginal fitness returns to increasing number of wives above and beyond the fitness costs of sharing a husband's wealth sharply limit the number of wives acquired by exceptionally wealthy individuals. We use our empirical data to demonstrate that the transition to agriculture is associated with both of these factors identified as drivers of monogamy.

### 1.1. The polygyny paradox

**Figure 1.**
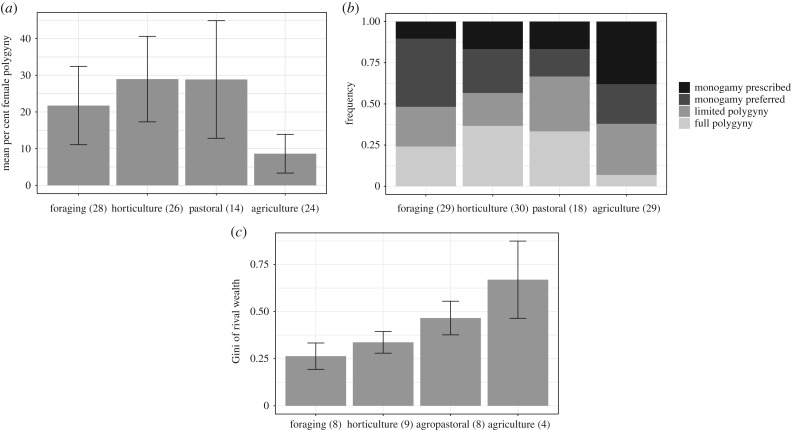
(*a*) Mean frequency of married women who are married polygynously by production system (±2 s.e.) using the Standard Cross-Cultural Sample [30]. Rates of polygyny are measured with variable ♯872, per cent of wives with co-wives. (*b*) Rates of monogamy and polygyny by production system are measured with variable ♯861, the standard polygamy code. Data on subsistence come from variable ♯858, categorized subsistence. In general, agricultural populations show reduced rates of polygyny and increased rates of monogamy relative to other subsistence systems. See electronic supplementary material for more information. (*c*) Gini of wealth by production system in our sample.

The Standard Cross-Cultural Sample [30] illustrates that the frequency of polygyny is relatively high in horticultural and pastoral populations, and low in agricultural populations. These findings are robust to use of quantitative (figure 1*a*) or qualitative (figure 1*b*) descriptors of polygyny. The third panel (figure 1*c*) presents our estimates of the extent of material wealth inequality among males in the four production systems.

Theoretical models of mating systems [1–4,31] predict that polygyny should be positively associated with inequality in male resources, and more specifically with what Murdock [32, pp. 206–207] terms ‘movable property or wealth which can be accumulated in quantity by men’. These forms of rival material wealth are, as we have just seen, more unequally held in horticultural economies than among foragers, which is consistent with the greater extent of polygyny in the former. Oh *et al.* [17] show that inequality in reproductively important, non-rival forms of wealth—network ties, genes conferring adaptive phenotypes or acquired knowledge, for example—can also be a strong driver of polygyny, contributing to the explanation of substantial levels of polygyny in some societies with little rival wealth inequality. Indeed, there is empirical evidence that non-rival forms of wealth are associated with polygynyous marriage in some foraging [33,34] and horticultural [35] populations.

While the polygyny threshold model has been effective in predicting the distribution of polygynous males within populations (e.g. [6,17]), the reduced level of polygyny in agricultural populations typically characterized by greater inequality poses a serious challenge to existing models of mating and marriage.

### 1.2. A comparative dataset

**Figure 2.**
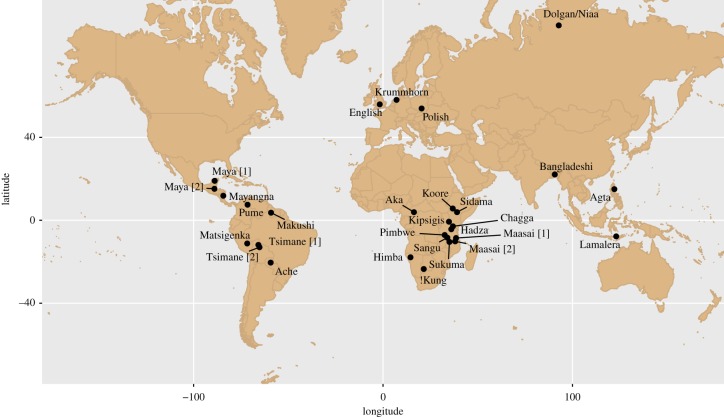
Locations of populations studied in this investigation (see table 1 for details).

To address this challenge, we build a comparative database of individual-level wealth, marriage and reproductive success measures in 29 diverse populations distributed over a wide geographical range (figure 2). Table 1 provides population-specific background data.

**Table 1.** Location, subsistence, marriage system and rival wealth proxies used in analysis of wealth inequality in 29 populations. Citations provide background information on the specified population. We acknowledge that most empirical wealth variables will lie somewhere on a continuum of rivalness, but we have attempted to choose variables that are more rival than non-rival for use in this empirical study. For more details on rival wealth and its comparability across sites, see the electronic supplementary materials. Note also, that we use the term *Mixed* to describe the mating system when concurrent marriage is socially accepted and practised alongside monogamy, but at lower intensity than is observed in more classical systems of polygyny—e.g. as among African agropastoralists. In groups with a mixed mating system, neither monogamy nor polygyny constitutes the sole form of marriage that is culturally obtainable or aspired towards. The sample size of males in each population is denoted by *N*.

ID	population	citation	location	subsistence	marriage system	rival wealth proxy	*N*
1	Aché	[36]	Paraguay	foraging	mixed	weight	117
2	Agta	[37]	Philippines	foraging	monogamy	weight	90
3	Aka	[38]	C.A.R.	foraging	mixed	weight	89
4	Dolgan/Niaa	[39]	Siberia	foraging	monogamy	territory, vehicles	308
5	Hadza	[40]	Tanzania	foraging	mixed	weight	100
6	!Kung	[41]	Botswana	foraging	monogamy	weight	81
7	Lamalera	[42]	Indonesia	foraging	monogamy	weight	238
8	Pumé	[43]	Venezuela	foraging	mixed	weight	46
9	Chagga	[44]	Tanzania	horticulture	monogamy	cows, land	49
10	Makushi	[45]	Guyana	horticulture	mixed	land	145
11	Matsigenka	[46]	Peru	horticulture	mixed	boats	37
12	Maya [1]	[47]	Belize	horticulture	monogamy	land	39
13	Maya [2]	[48]	Mexico	horticulture	monogamy	land, vehicles	85
14	Mayangna/Miskito	[49]	Nicaragua	horticulture	mixed	wealth	47
15	Pimbwe	[50]	Tanzania	horticulture	mixed	cows, land	231
16	Tsimane [1]	[51]	Bolivia	horticulture	mixed	wealth, land	250
17	Tsimane [2]	[52]	Bolivia	horticulture	mixed	wealth	263
18	Himba	[53]	Namibia	agropastoral	polygyny	cows, land	65
19	Kipsigis	[6]	Kenya	agropastoral	polygyny	cows, land	626
20	Koore	[54]	Ethiopia	agropastoral	monogamy	cows, land	82
21	Maasai [1]	[47]	Tanzania	agropastoral	polygyny	cows, land	57
22	Maasai [2]	[47]	Tanzania	agropastoral	polygyny	cows, land	133
23	Sangu	[55]	Tanzania	agropastoral	polygyny	cows, land	59
24	Sidama	[56]	Ethiopia	agropastoral	polygyny	cows, land	85
25	Sukuma	[57]	Tanzania	agropastoral	polygyny	cows, land	60
26	Bangladeshi (2000s)	[58]	Bangladesh	agriculture	mixed	land	1103
27	English (1800s)	[59]	England	agriculture	monogamy	wealth	3851
28	Krummhörn (1700s)	[60]	Germany	agriculture	monogamy	land	3106
29	Polish (1900s)	[61]	Poland	agriculture	monogamy	cows, land	371

In order to use all cohorts of the adult male populations, relevant measures—wives and wealth proxies—are age adjusted in a Bayesian framework to represent their predicted values at age 60. This method of age adjustment assumes that the additional acquisition of wives and wealth from the time of censor to the age of 60 are unmeasured positive random variables, with mean values governed by the remaining time for acquisition and the age-specific acquisition rate trajectories inferred from the population cross sections (see the electronic supplementary materials for mathematical details).

Our polygyny measures reflect the per cent of women who will ever be married to a man who marries more than once—in other words, in contrast to the data in figure 1*a*, we consider sequential marriage as a form of polygyny since the offspring of each mother are rival claimants to a father's property. Our age adjustment delivers a measure that could be called ‘completed polygyny’ by analogy to ‘completed fertility’. The populations exhibiting surprisingly high levels of polygyny by our definition—e.g. the Aché, Hadza, Maya, English and Krummhörn populations—reflect the prevalence of serial monogamy, not polygyny in the usual sense of multiple concurrent wives. Although most anthropological analyses of polygyny limit the definition of the term to two or more *co-occuring* wives of one man, we forego the sequential/concurrent distinction because (i) a male's wealth is generally shared to some degree across all wives and the children of each over the male's lifetime and (ii) as we show later, the elasticities^2^ of fitness with respect to times married are reliably positive for almost all populations sampled here, even those in which serial monogamy is practised. This suggests that males do indeed increase fitness through marriage to multiple women, even in cases in which these marriages are sequential.

Sequential marriage can be considered a form of polygyny insofar as men typically replace divorced wives with younger women, allowing a subset of males in the population to increase their lifetime reproductive success relative to less wealthy males in the population, as has been shown in many of the populations sampled here (e.g. [62–64]) and elsewhere, both directly [65] and indirectly [66]. The essential puzzle to be explained with our model, however, is not the extent to which effective polygyny is driven by concurrent marriage versus sequential remarriage, but rather how effective polygyny (i.e. inequality in wife-years and the resultant inequality in reproduction) can be attenuated by changes in the structuring of wealth inequality.

**Figure 3.**
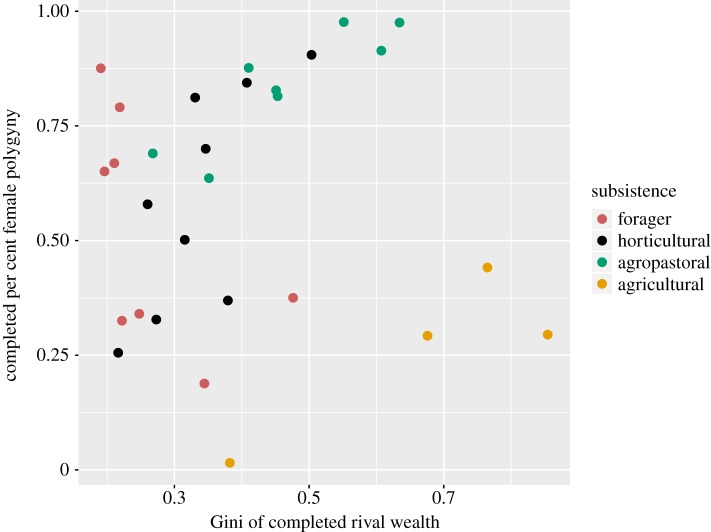
Relationship between the Gini coefficient on completed rival wealth and per cent completed female polygyny.

As in the Standard Cross-Cultural Sample [30], there is no overall relationship between wealth inequality as measured by the Gini coefficient and per cent age-adjusted female polygyny in our sample (figure 3). However, analysed by subsistence category, this relationship varies. Foragers show little variation in wealth inequality, but high variation in polygyny. Agricultural populations show high variation in inequality, but low and relatively invariant levels of polygyny. Among horticultural—*β* = 2.02 (90%CI: 0.65, 3.39)—and agropastoral—*β* = 0.86 (90%CI: 0.49, 1.24)—populations, we find the expected positive associations between wealth inequality as measured by the Gini coefficient and per cent female polygyny. A possible concern related to the cross-cultural compatibility of our estimates is that our rival wealth proxies vary between populations and productions systems; in cross-cultural projects as wide-ranging as this one, however, there is rarely a single variable that can be compared directly across populations—instead, we have relied on ethnographic accounts to identify which sources of wealth are most relevant to production and reproduction in each society, and attempted to build a cross-culturally comparable dataset by using the most locally relevant measures of wealth in each population (see electronic supplementary material for further discussion).

### 1.3. A mutual mate choice model of polygyny and its decline

Following Oh *et al.* [17], we consider a population of men with two types of fitness-relevant resources [67]: non-rival wealth, denoted as *g*, and rival wealth, denoted as *m*. As a useful mnemonic, we can think of *g* as the value of a male's genetic contribution to offspring production and survival, and *m* as the value of his material contributions, but the general definitions are considerably broader. Although we treat *g* and *m* as completely distinct stores of wealth in this mathematical model, we recognize that most empirical wealth variables will lie somewhere on a continuum of rivalness between non-rival resources, like genes, that can be provided to all offspring in equal measure, and rival resources, like land, which must be divided among offspring. For example, local ecological knowledge can be passed in equal measure to all offspring, but the time allocated to personal instruction may be rival. We represent the total mating investment devoted to acquiring a wife by a cost equal to *c* units of the rival resource per wife; this term includes classic costs, such as bride price, in addition to all other costs associated with courtship and marriage. For an explanation of these and all other variables and functions used in this paper, see table 2.

**Table 2.** Definitions of variables and functions.

symbol	domain	definition
*g*_p_	1	A poor male's non-rival wealth, like network ties or acquired knowledge. It may be passed in equal measure to all offspring of a given father.
*m*_p_	1	A poor male's rival wealth, like land. It must be divided among the offspring of a given father.
*g*_r_	(1, ∞)	A rich male's non-rival wealth. This value is defined in terms of units of *g*_p_.
*m*_r_	(1, ∞)	A rich male's rival wealth. This value is defined in terms of units of *m*_p_.
*c*	(0, *m*_p_)	The total mating investment (in units of the rival resource) devoted to acquiring a wife.
*γ*	(0, 1)	The percentage increase in male fitness associated with a 1% increase in the male's non-rival wealth per wife. We assume that *μ* + *γ* < 1.
*μ*	(0, 1)	The percentage increase in male fitness associated with a 1% increase in the male's rival wealth per wife. We assume that *μ* + *γ* < 1.
*δ*	(*μ*, 1)	The percentage increase in male fitness associated with a 1% increase in number of wives, holding constant total wealth per wife; if *δ* = 1, then doubling the number of wives (with a co-occurring increase in wealth sufficient to let the wealth per wife remain unchanged) will double male fitness. However, when *δ* < 1, there are diminishing fitness returns to additional wives, in addition to the fitness costs already associated with division of rival wealth across wives. We assume that *δ* > *μ* to ensure that the elasticity of fitness with respect to the number of wives is positive.
*θ*	(0, 1)	Frequency of rich males in a population. In the theoretical models, this is a defined parameter. In the empirical models, we estimate this parameter using: (i) the frequency of men in the upper *ϕ* percentile of cumulative wealth in each population and (ii) the frequency of men with more than *ψ* wealth, where *ψ* is the empirical level of rival wealth that on average separates men with two wives from men with a single wife. More details about these metrics are included in the electronic supplementary material.
*w*	(0, ∞)	Male fitness. See equation (1.1).
n^*^	(0, ∞)	Number of wives per rich male at Nash equilibrium. See equation (1.3).
*P*	(0, 1)	Per cent female polygyny (as per cent of wives with co-wives) at Nash equilibrium. See equation (1.4).
*s*	(0, ∞)	Number of males per female in the population.
*G*	(0, 1)	Gini coefficient. A measure of wealth inequality varying from 0—no inequality—to 1—one person has all of the wealth. In a population with two levels of wealth, low and high, with the high wealth group being *u*% of the population and holding a fraction *f*% of all wealth, the Gini coefficient is *f* − *u*. In our case, this would imply that the Gini of the wealth distribution is given by the function: *G*(*θ*, *m*_r_) = (*θm*_r_/(*θm*_r_ + (1 − *θ*))) − *θ*. In the case of a continuous distribution of wealth, the Gini is expressible as half the relative mean absolute difference in wealth values [68].

If a man marries *n* women, the non-rival wealth available to each wife is *g* and the rival wealth available to each wife is (*m* − *cn*)/*n*. As in Oh *et al.*'s [17] model, we assume that each wife produces offspring as a function of the wealth she has been provided by the male, adjusted for the importance—*γ* and *μ*—of each type of wealth to fitness. The fitness, *w*, of a male is then given by the effective number of wives acquired by the male multiplied by their average fitness:1.1
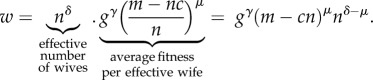
The parameters *γ* and *μ* are constrained to the unit interval reflecting the assumption—strongly confirmed in our empirical estimates—that the marginal fitness effect of additional wealth of either type, while positive, is either constant or diminishing as wealth increases. Note that rival and non-rival wealth are modelled as complementary inputs using a fitness function analogous to the Cobb–Douglas function widely used in economics. This assumption formalizes the idea that having high non-rival wealth (like farming skill) with limited material wealth (like land, seed and farming tools) will not contribute as much to fitness as having farming skill in the presence of substantial amounts of such material resources. In other words, the multiplicative nature of the fitness function means that the marginal fitness effect of each kind of wealth is greater as the amount of the other kind of wealth increases.

The parameter *δ* is key to our proposed resolution of the polygyny paradox. It controls the extent of diminishing returns to increasing number of wives for reasons unrelated to the need to share a male's rival wealth among wives; a value of one indicates no such sources of diminishing returns and an increasing extent of such diminishing returns is indicated by values of *δ* falling farther below one. In the model, as *δ* decreases the *effective number of wives* falls further below the empirically observed number *n*, indicating that female reproduction is constrained in some way by a male's additional marriages for reasons other than rival wealth sharing.

In order to produce analytically tractable results, we simplify by assuming throughout that there are only two types of males, rich and poor, with rich males being a fraction *θ* of the population. All rich are identical, as are all poor. The rich males are indexed by *r* and the poor by *p*. We also assume that females hold no wealth and are identical in their reproductive potential. Neither of these assumptions is essential to the model or its behaviour, and both can be relaxed via computational methods (e.g. as presented in Oh *et al.* [17]).

In the analysis that follows, females will maximize their reproductive success by becoming the *n*th wife of a rich male so long as the following condition—our generalized variant of the polygyny threshold, accounting for both rival and non-rival wealth and the relative importance of each—is satisfied:1.2
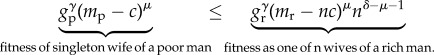


Equations (1.1) and (1.2) determine the fitness of males and females. Without loss of generality, we can define *m*_r_, *m*_p_ and *c* in terms of units of *m*_p_, and likewise with *g*_r_ and *g*_p_, so that *m*_r_, for example, means the rival wealth of a ‘rich’ man relative to the rival wealth of a ‘poor’ man, and analogously for non-rival wealth.

At any moment in time, there may be women seeking a polygynous marriage and not finding a suitor, or the opposite, men seeking but not finding additional wives. In the first case—borrowing terms from economics—we say that ‘male demand is limiting’ and in the second that ‘female supply is limiting’. It is also possible for both male demand and female supply to be jointly limiting, specifically in ‘market clearing’ cases—defined as those cases where supply exactly matches demand. Economic models of martial matching generally assume that the bride price, *c*, will adjust to either excess supply (i.e. under which *c* will decrease) or excess demand (under which *c* will increase), to generate this market clearing equilibrium. Our model and our explanation for the decline of polygyny with increasing inequality, however, apply independently of whether or not the market clears.

From equation (1.1), we derive (see [17]) an analytic expression for the equilibrium number of wives desired by each rich man, n^*^:1.3
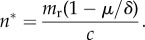
As one would expect, male demand for wives is larger when (i) the rich class of males have greater average wealth than the poor class of males (large *m*_r_), (ii) the mating cost is low (small *c*), (iii) the importance of rival wealth as a determinant of fitness realized through wives and their children is low (small *μ*) and (iv) diminishing fitness returns to additional wives—for reasons independent of the need to share rival wealth—are low (large *δ*). Equation (1.3) yields the number of wives that each rich man has if male demand is limiting, or if the cost of bride price and mating investments, *c*, is such that the market clears. However, if female supply is limiting, and the market does not clear, then there is no simple closed form solution for n^*^.

We distinguish between two different measures of polygyny in a population—n^*^, the number of wives of each rich man at equilibrium, and *P*, the percentage of wives with co-wives at equilibrium. If at least some women marry monogamously, and male demand is limiting, then there is a closed form solution for *P* at equilibrium (see electronic supplementary material), given by:1.4
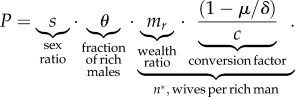
Note here that n* might be large even when *P* is small if there are few rich men in the population. Historic Greek and Roman marriage systems have been described in just this way [69,70]. In these societies, up to two-thirds of the population (including slaves) had almost no significant forms of material wealth [15]—sharply limiting the ability of the majority of the male population to marry polygynously.

From equation (1.4), we see that there are distinct effects of the two components of the male wealth distribution—(i) the frequency of rich males in the population, *θ* and (ii) the relative wealth of the rich and poor males, *m*_r_—on the population-level frequency of wives with co-wives, *P*. The final term (conversion factor) on the right of equation (1.4) controls the extent to which a rich man's elevated share of rival wealth, *m*_r_ is convertible into an elevated share of wives. The extent to which wealth differentials can be transformed into differentials in wife number will depend on the importance of rival wealth in the fitness equation, *μ*, the extent of diminishing fitness returns to increasing number of wives, *δ*, and the mating investment costs, *c*.

## 2. Results

### 2.1. Theoretical results

We now demonstrate two theoretical results with the potential to resolve the polygyny paradox. First, diminishing returns to additional wives arising from causes other than necessity to share a husband's rival material wealth will reduce the number of wives acquired by each rich male. Second, because of this fact, a highly unequal wealth distribution with few extraordinarily rich men may produce little polygyny, while a less unequal wealth distribution with a larger fraction of rich men may produce a greater extent of polygyny. Two rich men, for example, can be expected to have more wives in total than one very rich man whose wealth equals their combined wealth. For this same reason, the Gini coefficient—see table 2 for a definition—is not a sufficient statistic for the analysis of the relationship between polygyny and wealth inequality. We take up each of these results, in turn, before assessing if our empirical estimates are consistent with this explanation.

#### 2.1.1. Decreasing fitness returns to increasing number of wives reduce how many wives are acquired by rich males

If we assume that male demand is limiting, then equation (1.3) determines the number of wives each rich man will have. It is clear from inspection of equation (1.3) that a greater extent of diminishing fitness returns to additional wives (i.e. a lower value of *δ*) produces a lower male demand for additional wives. This is demonstrated mathematically in the electronic supplementary material.

Determining the effect of greater diminishing returns to additional wives (i.e. a lower value of *δ*) when female supply is limiting is more challenging. As noted above, if female supply is limiting, the value of n* implied by the polygyny threshold inequality in equation (1.2) has no closed form solution. To address this challenge, we proceed as follows. Suppose that the polygyny threshold in equation (1.2) were satisfied by an equality. Then a reduction in *δ*, holding all other terms constant, would reduce the right-hand side of the equation—the fitness of each of the *n* wives of a rich man—while having no effect on the left-hand side—the fitness of a singleton wife. Thus, holding all else equal, an offsetting decrease in *n* would be required to restore the equality. This is demonstrated in the electronic supplementary material by differentiating equation (1.2) with respect to *δ*.

This means that a man who was just barely rich enough so that an unpaired woman would choose to marry him as wife number (*n* + 1) under the initial *δ*, would, under the lower *δ*, be unable to secure the unpaired woman's partnership. As such, an increase in the extent of diminishing returns to additional wives (lower *δ*) will reduce both male demand for, and female supply to, polygynous marriage.

Our results imply that if *δ* < 1, a larger quantity of moderately rich men can be expected to have more wives in total than a smaller quantity of even richer men, holding constant the total wealth held by the rich across these cases. This first finding will interact with our second finding, discussed below, concerning the effects of the population density of the rich class of men on the frequency of polygyny and the level of wealth inequality. In the electronic supplementary material, we present an alternative approach to account for diminishing marginal returns to increasing number of wives and find that our insights do not depend on the specific way in which diminishing fitness returns to increasing number of wives are modelled.

#### 2.1.2. Highly concentrated wealth inequality can reduce the frequency of wives with co-wives

The structuring of rival wealth across men has implications for both per cent female polygyny and the Gini coefficient of rival wealth. In short, to analyse the relationship between wealth inequality and per cent female polygyny, we need more information than can be provided by the Gini coefficient alone.

**Figure 4.**
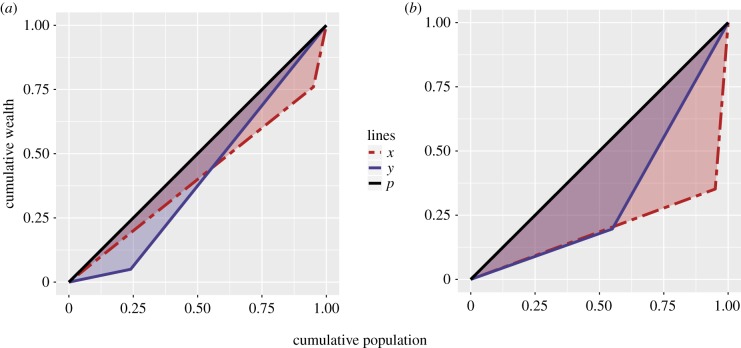
Lorenz curves of hypothetical wealth distributions. Points on the Lorenz curves represent statements like: ‘the bottom *j*% of all males have *k*% of the total wealth.’ In both subfigures, the diagonal line *p* represents an equal distributional of wealth. In this case, the bottom *j*% of males have exactly *j*% of the wealth. As inequality grows, the shaded area between the Lorenz curves (i.e. *x* or *y*) and the line of perfect equality, *p*, expands. This area, multiplied by 2, is equal to the Gini coefficient. Details of the *x* and *y* wealth distributions, as well as details concerning the level of polygyny supported by each, are discussed in the main text. (*a*) Lorenz curves with fixed Gini coefficients and wealth ratios, but differing per cent rich and (*b*) Lorenz curves with differing Gini coefficients, wealth ratios and per cent rich.

Consider the two Lorenz curves in figure 4*a*. The Gini coefficient (0.19) and wealth ratio (*m*_r_/*m*_p_ = 6) for the two distributions are identical, but wealth is distributed differently. In the case of *x*, inequality arises from a large fraction of wealth being held by a small class of very rich elite (*θ* = 0.05). In the case of *y*, however, inequality arises from the presence of a moderately sized class of relatively poor individuals (*θ* = 0.76). Under the assumptions of our model, these wealth distributions differ in a way that is critical to the fraction of women that will be polygynously married.

To see the implications that each of the above wealth distributions has for the extent of polygyny, we use the theoretical model given by equation (1.4). If we set *s* = *c* = *m*_p_ = 1, *μ* = 0.4, and *δ* = 0.5, for example, and assume that male demand is limiting, we can calculate the percentage of wives with co-wives under each wealth distribution. Note that this value of *μ* is much greater than the mean of our empirical estimates, 0.08, (90%CI: 0.05, 0.11). In this example, rival wealth is of substantial importance for fitness and this is expected to act as a major impediment to polygyny; but even under these parameters, the wealth distribution in *y* is sufficient to drive polygyny to fixation (*p* = 1.00, all wives have co-wives). By contrast, the wealth distribution in *x* generates little polygyny (*p* = 0.10) at the same levels of *c*, *μ* and *δ*. The distribution with few rich men supports less polygyny than the distribution with a greater number of rich men.

The same idea holds true in figure 4*b*, which illustrates a more empirically plausible scenario; here the *x* distribution, coloured red and representing a hypothetical agricultural population, is characterized by a very large rich-to-poor wealth ratio (*m*_r_/*m*_p_ = 35) and Gini coefficient (0.60), with wealth still being concentrated by a small fraction of rich elite (*θ* = 0.05). The *y* distribution, coloured blue and representing a hypothetical horticultural population, is characterized by a more modest rich-to-poor wealth ratio (*m*_r_/*m*_p_ = 5) and Gini coefficient (0.35), with wealth being concentrated by a larger fraction of rich (*θ* = 0.45). If we again set *s* = *c* = *m*_p_ = 1, *μ* = 0.4 and *δ* = 0.5, and assume that male demand is limiting, we find that the wealth distribution in *y* is sufficient to drive polygyny to higher levels (*p* = 0.45) than in *x* (*p* = 0.35), even though wealth inequality in *x* is *much* greater than in *y*.

To anticipate our empirical analysis: we find that agricultural wealth distributions are more like *x* in the example from figure 4*b* (large Gini, large wealth ratio and small per cent rich), and horticultural wealth distributions are more like *y* (moderate Gini, moderate wealth ratio and moderate per cent rich). For agricultural-like wealth distributions with diminishing fitness returns to increasing number of wives (*δ* < 1), large differences in relative wealth cannot compensate for the smaller number of rich men in the production of polygyny, because doubling a male's wealth will not double his expected number of wives.

Importantly, an increase in the population density of poor individuals can have directionally opposite effects on the rival wealth Gini and the extent of polygyny. For example, an increase in the population density of poor individuals, (1 − *θ*), from say a growing class of equally disadvantaged peasants, can have the effect of decreasing per cent female polygyny, *P*, while increasing inequality in the distribution of rival wealth as measured by the Gini coefficient. The first point is apparent simply from noticing that *θ* (the frequency of rich males) is a factor in the production of *P* in equation (1.4). The second point can be demonstrated by showing that changing *θ* does not have a monotonic effect on wealth inequality. In particular, for large *m*_r_ (the rich-to-poor wealth ratio) decreasing *θ* generally increases wealth inequality. We demonstrate this by showing that the partial derivative of the Gini coefficient (table 2) with respect to *θ* is negative whenever:2.1
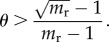
The above equation suggests that over a range of *θ*, a small decrease in *θ* will lead to an increase in wealth inequality as measured by the Gini coefficient. In fact, the right-hand side of equation (2.1) goes to zero as *m*_r_ → ∞. See the electronic supplementary material for mathematical details and a visualization of the range of parameters sufficient to generate this effect. We also show in the electronic supplementary material that the ratio of per cent female polygyny to the Gini coefficient on rival wealth is monotonically decreasing as the fraction of rich males declines.

A population can have a very large Gini coefficient for rival wealth, but if that inequality is driven by the existence of a large population of landless peasants, for example, it will be structured in such a way that it produces lower population levels of polygyny (as in figure 4*b*). The relationship between wealth inequality and polygyny cannot be fully understood by investigating cross-cultural variation in the Gini of rival wealth. More finely resolved models are needed to disentangle the countervailing effects of *θ* and *m*_r_ on population-level marriage outcomes.

### 2.2. Empirical results

#### 2.2.1. Predictions

If changes in the level of wealth inequality that accompanied the transition from relatively egalitarian horticulture to socially stratified agriculture were driven strongly by the population growth of a large class of poor peasants—i.e. decreasing *θ*—and if there are diminishing fitness returns to increasing number of wives beyond those entailed by the sharing of the male's rival wealth—i.e. *δ* < 1—then we would expect to see a decline in the frequency of polygyny with the rise of agriculture.

Specifically, we predict:

(P1) Empirical estimates of equation (1.1) will show that *δ* is less than 1 in most human populations. This provides evidence of diminishing fitness returns to additional wives for reasons other than the sharing of the male's rival wealth.
(P2) Empirical estimates of *θ* will be smaller in agricultural economies than in other subsistence systems. This provides evidence of a decreasing fraction of males capable of meeting the polygyny threshold in agricultural populations.

#### 2.2.2. Estimates of fitness elasticities

**Figure 5.**
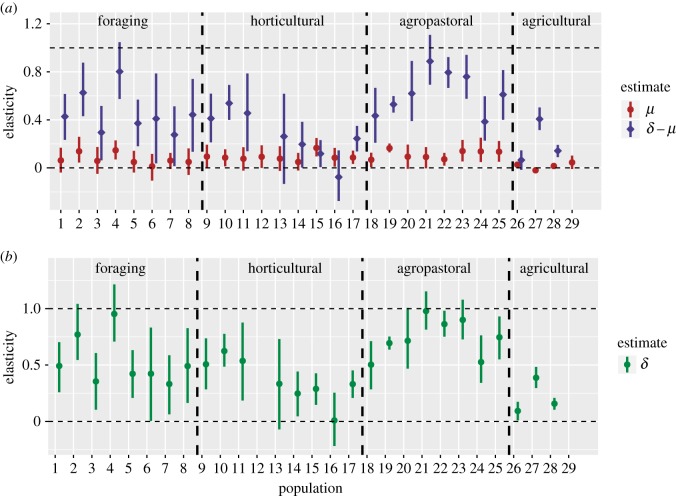
Empirical estimates of the elasticity of reproductive success on wives and rival wealth. The elasticity of fitness on wives is estimated using a parameter representing the value: *δ* − *μ*. We simply add our estimate of *μ* to this value to yield an estimate of *δ*. We find that *δ* is typically much less than 1, but also reliably non-zero. Note that posterior estimates of *μ* and *δ* − *μ* (in red and blue) are paired by population ID along the *x*-axis; two populations—12 and 29—have missing estimates of *δ* − *μ* and *δ* because in these populations all males had only a single wife. See table 3 for population names. (*a*) Empirically estimated fitness elasticities on rival wealth, *μ*, and wives, *δ* − *μ* and (*b*) implied value of diminishing returns to increasing wife number for reasons unrelated to rival wealth sharing, *δ*; a lower value of *δ* signifies greater diminishing returns.

To address prediction P1, we present empirical estimates of *μ* (the importance of rival wealth) and *δ* (the extent of diminishing return to increasing number of wives for reasons other than rival wealth sharing) (figure 5). These values are estimated using a multi-level regression model fit to our individual-level data; methodological details are provided in the electronic supplementary material. In all but four of the populations in our sample, the estimated *δ* coefficient is reliably less than 1. This result provides cross-cultural empirical support for the first of the two conditions needed to generate a transition to a greater degree of monogamy with increasing wealth inequality. Note two further results also shown in figure 5. First, our estimates for *μ* are quite low, particularly across the agricultural economies. Second, our estimates of *δ* − *μ* are positive in almost all populations, including those that are concurrently polygynous and those that are serially monogamous.

**Table 3.** Posterior wealth and polygyny estimates. Values are medians and 90% credibile intervals. The estimates of proportion rich and wealth ratio presented here are calculated using the minimum frequency of men that own 50% of the population's total wealth, after adjusting for age. Our age-adjustment methodology is detailed in the electronic supplementary material. Estimates of polygyny (as per cent of wives with co-wives) are also adjusted for age. The final variable polygyny-60 is a calculation of the empirically observed frequency of wives with co-wives among men age 60 or above. Our use of the term *co-wives* refers to the frequency of women married to a man who has married or will marry more than once. To calculate these values, however, we are limited to using only a small subset of the data in each population taken from men age 60 and above. This variable serves as a check on our age-adjustment methodology; we observe a strong positive correlation between the completed polygyny estimates and the observed completed polygyny data, *ρ* = 0.93. The bottom block of estimates are means by subsistence type.

ID	population	subsistence	wealth Gini	wealth ratio	proportion rich	polygyny	polygyny-60
1	Aché	forager	0.19 (0.18, 0.24)	1.7 (1.6, 2.0)	0.37 (0.33, 0.38)	0.66 (0.55, 0.74)	0.8
2	Agta	forager	0.24 (0.24, 0.27)	1.9 (1.9, 2.1)	0.33 (0.31, 0.34)	0.34 (0.27, 0.42)	0.3
3	Aka	forager	0.18 (0.17, 0.24)	1.7 (1.6, 1.9)	0.37 (0.34, 0.38)	0.88 (0.81, 0.93)	0.75
4	Dolgan/Niaa	forager	0.47 (0.46, 0.50)	5.2 (4.6, 6.1)	0.16 (0.14, 0.18)	0.38 (0.26, 0.48)	0.11
5	Hadza	forager	0.20 (0.20, 0.26)	1.7 (1.7, 2.0)	0.36 (0.32, 0.36)	0.67 (0.56, 0.76)	0.68
6	Kung	forager	0.22 (0.21, 0.26)	1.8 (1.8, 2.1)	0.35 (0.32, 0.36)	0.33 (0.08, 0.56)	0.18
7	Lamalera	forager	0.34 (0.32, 0.37)	2.8 (2.6, 3.0)	0.26 (0.24, 0.28)	0.19 (0.10, 0.29)	0.14
8	Pumé	forager	0.21 (0.20, 0.28)	1.8 (1.7, 2.2)	0.35 (0.30, 0.37)	0.79 (0.70, 0.88)	0.5
9	Chagga	horticultural	0.25 (0.24, 0.37)	2.0 (1.9, 3.0)	0.33 (0.24, 0.33)	0.32 (0.18, 0.48)	0.17
10	Makushi	horticultural	0.33 (0.24, 0.40)	2.6 (2.0, 3.2)	0.28 (0.23, 0.33)	0.82 (0.68, 0.92)	–
11	Matsigenka	horticultural	0.40 (0.34, 0.52)	3.7 (2.5, 5.2)	0.19 (0.14, 0.27)	0.85 (0.71, 0.94)	1
12	Maya [1]	horticultural	0.38 (0.27, 0.46)	3.3 (2.2, 4.7)	0.23 (0.15, 0.31)	0.39 (0.00, 0.73)	–
13	Maya [2]	horticultural	0.22 (0.21, 0.23)	1.9 (1.8, 2.0)	0.34 (0.32, 0.35)	0.26 (0.07, 0.46)	0.11
14	Mayangna	horticultural	0.51 (0.39, 0.59)	5.6 (3.2, 8.3)	0.15 (0.11, 0.23)	0.91 (0.82, 0.96)	0.93
15	Pimbwe	horticultural	0.33 (0.27, 0.47)	2.7 (2.1, 4.7)	0.27 (0.17, 0.32)	0.70 (0.65, 0.76)	0.69
16	Tsimane [1]	horticultural	0.26 (0.24, 0.29)	2.1 (2.0, 2.3)	0.32 (0.30, 0.33)	0.58 (0.46, 0.69)	0.56
17	Tsimane [2]	horticultural	0.31 (0.28, 0.36)	2.4 (2.2, 2.8)	0.29 (0.26, 0.31)	0.50 (0.38, 0.61)	0.55
18	Himba	agropastoral	0.65 (0.52, 0.71)	9.6 (4.6, 13.9)	0.09 (0.06, 0.17)	0.98 (0.96, 0.99)	0.96
19	Kipsigis	agropastoral	0.45 (0.43, 0.47)	4.0 (3.7, 4.3)	0.20 (0.19, 0.21)	0.83 (0.80, 0.86)	0.89
20	Koore	agropastoral	0.35 (0.32, 0.39)	2.9 (2.5, 3.4)	0.26 (0.22, 0.28)	0.64 (0.46, 0.79)	0.25
21	Maasai [1]	agropastoral	0.61 (0.55, 0.66)	8.0 (6.0, 11.3)	0.11 (0.07, 0.14)	0.92 (0.86, 0.96)	0.94
22	Maasai [2]	agropastoral	0.55 (0.51, 0.61)	6.4 (5.5, 8.2)	0.14 (0.11, 0.15)	0.98 (0.96, 0.99)	0.99
23	Sangu	agropastoral	0.44 (0.38, 0.58)	4.2 (3.2, 10.0)	0.19 (0.08, 0.24)	0.82 (0.72, 0.89)	0.67
24	Sidama	agropastoral	0.26 (0.24, 0.31)	2.2 (2.0, 2.5)	0.31 (0.28, 0.33)	0.69 (0.54, 0.82)	0.6
25	Sukuma	agropastoral	0.41 (0.37, 0.46)	3.3 (2.9, 3.9)	0.22 (0.20, 0.25)	0.88 (0.82, 0.92)	0.86
26	Bangladesh	agricultural	0.69 (0.59, 0.72)	10.3 (6.1, 12.1)	0.09 (0.08, 0.14)	0.29 (0.25, 0.34)	0.28
27	English	agricultural	0.86 (0.79, 0.89)	37.3 (15.1, 47.3)	0.03 (0.02, 0.06)	0.29 (0.28, 0.32)	0.25
28	Krummhörn	agricultural	0.77 (0.71, 0.79)	11.9 (9.0, 12.6)	0.08 (0.07, 0.10)	0.44 (0.42, 0.46)	0.43
29	Polish	agricultural	0.37 (0.37, 0.42)	3.0 (3.0, 3.3)	0.25 (0.23, 0.25)	0.01 (0.00, 0.04)	0
		forager	0.26 (0.25, 0.28)	2.4 (2.2, 2.5)	0.32 (0.30, 0.32)	0.53 (0.48, 0.57)	0.40
		horticultural	0.34 (0.31, 0.36)	3.0 (2.6, 3.5)	0.26 (0.24, 0.28)	0.59 (0.53, 0.65)	0.56
		agropastoral	0.47 (0.44, 0.49)	5.2 (4.4, 6.1)	0.19 (0.17, 0.20)	0.84 (0.81, 0.87)	0.88
		agricultural	0.67 (0.64, 0.69)	15.4 (10.0, 18.4)	0.11 (0.10, 0.13)	0.26 (0.25, 0.27)	0.27

The consistently small values of *μ* across all of our samples, even the monogamous ones, was unexpected. However, these low values reflect changes in male fitness *per wife*. Because of biological limits to the rate of reproduction in human females, significant increases to wealth are constrained to have less than proportional effects on fitness per wife. The effects observed here are more likely to reflect the ability of males with more than a threshold level of resources per wife to minimize offspring mortality [71,72], rather than to significantly enhance their own fertility. Though not discussed in detail here, our data suggest that male wealth impacts male fitness primarily by increasing the rate of wife acquisition rather than by increasing reproductive success per wife (see also, [73]).

Our second point addresses the possible concern that our estimates of *δ* may be low, in part, because we use *times married* as our measure of polygyny. While it is true that men can accumulate a greater maximal number of marriage years through concurrent polygyny than serial monogamy, figure 5*a* demonstrates that the use of times married is an appropriate measure of polygyny for our purposes. Across almost all populations, the elasticity of fitness with respect to times married, *δ* − *μ*, is positive and reliably non-zero. Because these estimates measure the population-specific effects of cumulative number of wives on reproductive success (see the exponent on *n* in equation (1.1)), they demonstrate that an increased number of marriages leads to increased reproductive success in both types of marriage systems—concurrently polygynous and serially monogamous.

#### 2.2.3. Estimates of per cent rich

We have established that there exists a strong cross-cultural pattern of decreasing—but reliably non-zero—fitness returns to increasing number of wives for reasons beyond rival wealth sharing. We now turn our attention to testing if the transition to agriculture is associated with a decreasing fraction of wealthy males.

In our theoretical model, we assume a discrete two-class wealth distribution, but empirical wealth data typically have continuous distributions. To deal with this issue, we consider two proxy measures for per cent rich in our empirical data: (i) the minimum percentage of men that account for a fraction *ϕ* of the total wealth and (ii) the frequency of men with more than *ψ* wealth, where *ψ* is the empirical midpoint in each population between the average wealth of males with one wife and the average wealth of males with two wives. More details about these metrics are included in the electronic supplementary material. Table 3 provides population-level posterior estimates of the completed wealth and completed polygyny measures, with the mean estimates by subsistence type shown in the bottom panel.

**Figure 6.**
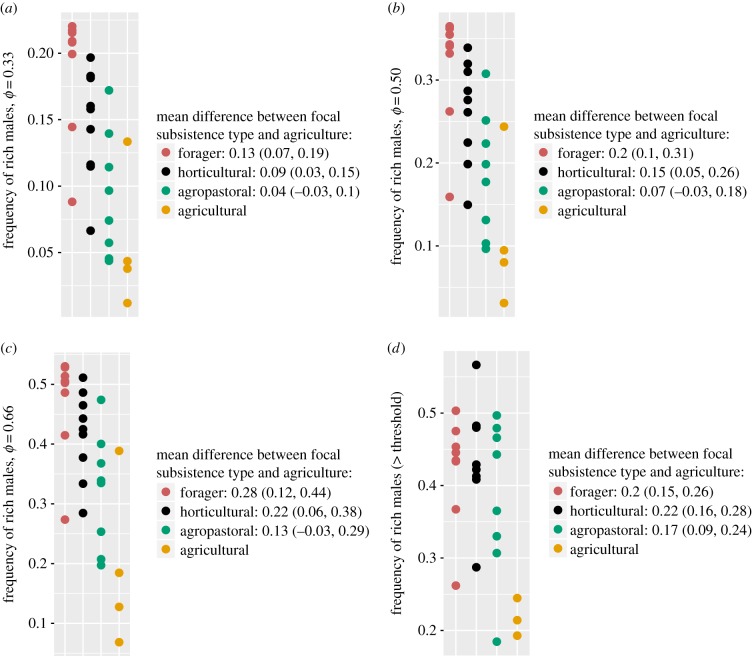
Frequency of rich males. Frames (*a*), (*b*) and (*c*) illustrate the minimal fraction of men who possess the upper *ϕ* fraction of cumulative wealth in the population. We see that wealth in agricultural populations is disproportionately possessed by a significantly smaller fraction of the population than in horticultural or even agropastoral societies. In frame (*d*), we calculate the empirical fraction of men with sufficient wealth to take on multiple wives, assuming the 2-polygyny threshold to rest halfway between the average wealth for men with one wife and the average wealth of men with two wives. Our sample size is reduced by two populations—12 and 29—in frame (*d*) because in these populations all males had only a single wife. Values listed in the legends show the mean difference (and 90% confidence intervals from a *t*-test) in the frequency of rich males between the focal subsistence type and agricultural populations. For example, in frame (*a*) the estimate of the mean frequency of rich males in horticultural populations was 0.09 (90%CI: 0.03, 0.15) higher than the corresponding mean estimate in agricultural populations. Per cent of men with (*a*) greater than or equal to 0.33 of total wealth, (*b*) greater than or equal to 0.50 of total wealth, (*c*) greater than or equal to 0.66 of total wealth and (*d*) greater than or equal to empirical 2-polygyny threshold.

To address prediction P2, we calculate empirical estimates of the fraction of rich men by production system (figure 6). We find that agricultural populations have a significantly reduced frequency of wealthy individuals relative to horticultural populations. All four panels show reliable differences in mean per cent rich between the horticultural and agricultural subsistence modes. This lesser fraction of wealthy individuals suggests a decreased number of men both able and willing to take second wives. This in turn leads to reduced levels of per cent female polygyny in contexts where large wealth differentials are not able to underwrite large differentials in wives due to the existence of diminishing fitness returns to such additional wives.

A limitation of this last result is that it is based on data from only four agricultural populations, three of them concentrated in a restricted region and time period (table 1). Moreover, a more informative dataset would come not from agricultural populations in the time period between the 1700s and 2000s, but rather from the agricultural populations in which monogamy actually began to emerge *de novo*. In our main analysis, we use estimates derived from the individual-level records available in the populations shown in table 1; in the electronic supplementary material, we present comparable analyses that include 14 additional wealth distributions from historical agricultural populations. The results of this supplementary analysis are consistent with our arguments here—and in fact show stronger and more reliable effects in the direction predicted by P2. These supplementary data, however, are based on sometimes contested reconstructions of the historical wealth distributions pieced together by archaeologists and economic historians; they must be appreciated within the constraints associated with such forms of data.

#### 2.2.4. Summary of empirical results

Using individual-level data from 29 populations, we show evidence of a general cross-cultural pattern of decreasing marginal fitness returns to increasing number of marriages. Further, using these same 29 datasets (reinforced in the electronic supplementary material with 14 additional wealth distributions from historical agricultural populations), we demonstrate the existence of an increasingly skewed distribution of material wealth in class-based agricultural societies (i.e. more of the wealth is concentrated in a smaller, elite fraction of the population). Both of these empirical findings are consistent with our model-based explanation for the decline of polygyny in societies engaged in agricultural production.

## 3. Discussion

We use cross-cultural data and a new mutual mate choice model to propose a resolution to the polygyny paradox. Following Oh *et al.* [17], we extend the standard polygyny threshold model to a mutual mate choice model that accounts for both female supply to, and male demand for, polygynous matchings, in the light of the importance of, and inequality in, rival and non-rival forms of wealth. The empirical results presented in figures 5 and 6 demonstrate two phenomena that are jointly sufficient to generate a transition to more frequent monogamy among populations with a co-occurring transition to a more unequal, highly stratified, class-based social structure. In such populations, fewer men can cross the wealth threshold required to obtain a second wife, and those who do may be fabulously wealthy, but—because of diminishing marginal fitness returns to increasing number of marriages—do not acquire wives in full proportion to their capacity to support them with rival wealth. Together, these effects reduce the population-level fraction of wives in polygynous marriages.

Our model demonstrates that a low population-level frequency of polygyny will be an equilibrium outcome among fitness maximizing males and females in a society characterized by a large class of wealth-poor peasants and a small class of exceptionally wealthy elite. Our mutual mate choice model thus provides an empirically plausible resolution to the polygyny paradox and the transition to monogamy which co-occurred with the rise of highly unequal agricultural populations.

We, however, cannot yet explain the causes of the unexpectedly substantial decreasing marginal fitness returns to increasing number of marriages. A purely statistical explanation of our results could be that we have missed some important rival form of wealth, which if accounted for would result in a larger estimate for *μ* and hence a reduced estimate of the degree of diminishing returns to additional wives for reasons other than the sharing of rival wealth. Another possibility, already mentioned, is that in some of our datasets the very wealthy could be deliberately limiting their reproductive success (i.e. passing through the demographic transition), which would also drive *μ* downwards.

In addition to these possible statistical effects, there are a number of other plausible causes of the diminishing returns to additional wives observed in our populations. One possibility is that a male's time and attention are rival inputs to his own fitness. This situation is likely to arise when paternal investment is essential to offspring survival and well-being. A male's time can also be rival in other fitness relevant ways. For example, it may be difficult for a single wealthy man to effectively mate guard a large number of wives. With a wealth ratio of *m*_r_ = 2 and a per cent rich of *θ* = 0.5, a single rich man will have to monopolize his two wives in the face of challenges from a single unmarried man on average; however, with a wealth ratio of *m*_r_ = 10 and a per cent rich of *θ* = 0.1, a single rich man will have to defend his 10 wives from nine unmarried men on average. As the wealth ratio grows even more skewed, this situation could become increasingly difficult to manage (e.g. requiring the use of eunochs to defend harems [74]).

A related possibility is that a growing number of unmarried men could socially censure wealthy polygynous males, imposing costs on them that reduce male demand for and/or female supply to polygynous marriage [23,24].

A third possibility is that sexually transmitted infection (STI) burden [22,75] could diminish returns to polygyny, if polygyny enhances infection rates [76,77]. Imagine that an STI which leads to infertility occurs in the female population with probability *κ*. Then, an uninfected man and his wives can avoid infertility if and only if none of his wives have the infection. This occurs with probability: (1 − *κ*)^*n*^. If we assume an empirically plausible rate for this STI, like 0.07 (which is lower than the contemporary prevalence of curable STIs in Western Europe [78]), then a monogamous man would be paired with fertile woman 0.93 of the time, and a man in 2-polygyny would be paired with fertile women 0.86 of the time. An ultra-wealthy man in 10-polygyny, however, would be more likely than not (0.52) to have infertile wives—a prospect that could lead to diminishing fitness returns to, and hence, demand for, additional wives. A similar argument holds even if marriage is considered sequentially—as in serial monogamy—though the effect would be smaller.

Finally, impediments to cooperation or even outright conflict among co-wives can be greater as the number of wives increases. Interference competition among co-wives could impose significant fitness costs in settings where effective child rearing benefits from cooperation [79,80]. It could well be that incumbent wives resist incorporation of additional wives to the wealth sharing pool, perhaps with greater effectiveness as their numbers grow.

Empirically exploring these and other possible explanations for the unexpectedly substantial diminishing fitness returns to additional wives that are not explainable by the division of rival wealth among wives would be a valuable next step, but one that would take us beyond the formal modelling, database and comparative statistical methods that we have presented.

## 4. Conclusion

Building on the original polygyny threshold model [1,2], we have proposed—and provided some empirical support for—a new explanation for the decline of polygyny in highly stratified societies where rival wealth is concentrated by a small class of rich elite. To increase the scope and explanatory power of the original model, we have generalized it to include (i) mutual mate choice dynamics [28] and (ii) a consideration of both the level and structuring of wealth inequality. The two wealth types in our model are the end points on a continuum from rival to non-rival and we omit variability in the quality of females. Further, our demonstration is limited to a two-class wealth system.

To test our model, we have used individual-level data on wealth, marriage and fitness taken from 29 human populations. This allows us to estimate effects that cannot be calculated from population-level summaries in the Standard Cross-Cultural Sample or related databases. Across the majority of these populations we find strong evidence of (i) diminishing fitness returns to additional wives beyond those which occur due to the rival nature of wealth that is shared among wives, as well as (ii) a lesser fraction of wealthy men in highly unequal agricultural production systems. These data provide initial support for our proposed resolution of the polygyny paradox, and point to the benefits of integrating formal models of the relationship between inequality and mating system evolution with cross-cultural, individual-level databases.

More generally, we show that a mutual mate choice analysis of the fitness consequences of individual mating decisions offers a lens through which we can study the coevolutionary processes contributing to the transformation of marriage system norms. Some previous studies have addressed how social norms for monogamy might have plausibly spread post-emergence. Our findings provide an explanation for how monogamy can emerge *de novo*, even among fitness maximizing agents in highly unequal social contexts.

## Supplementary Material

Appendix

## Endnotes

1 In the parlance of economists, *rival* refers to forms of wealth, like land, that must be divided amongst a set of individuals—the offspring of a given father, for example. By contrast, *non-rival* wealth, like genes conferring a beneficial phenotype, can be passed in equal measure to all offspring of a given father. What economists term public goods are non-rival in this sense. Biologists writing about the same phenomena use the directly analogous terms *depreciable* and *non-depreciable* [16] to classify the forms of parental investments in offspring.

2 An elasticity is defined as the ratio of the percentage change in one variable to the percentage change in another—it is a unitless measure of the relationship between variables. In empirical work, if both dependent and independent variables are measured in natural logs, then the estimated regression coefficients from a linear model will be interpretable as elasticities.

## Data accessibility

Interested readers can replicate our analysis using the model code and data included with this submission. This code and database will be maintained at: https://github.com/ctross/publications/polygynypuzzle. Analysis is conducted using R [81] and RStan [82].
