# Correction to: ‘Greater wealth inequality, less polygyny: rethinking the polygyny threshold model’

**DOI:** 10.1098/rsif.2018.0752

**Published:** 2018-10-31

**Authors:** Cody T. Ross, Monique Borgerhoff Mulder, Seung-Yun Oh, Samuel Bowles, Bret Beheim, John Bunce, Mark Caudell, Gregory Clark, Heidi Colleran, Carmen Cortez, Patricia Draper, Russell D. Greaves, Michael Gurven, Thomas Headland, Janet Headland, Kim Hill, Barry Hewlett, Hillard S. Kaplan, Jeremy Koster, Karen Kramer, Frank Marlowe, Richard McElreath, David Nolin, Marsha Quinlan, Robert Quinlan, Caissa Revilla-Minaya, Brooke Scelza, Ryan Schacht, Mary Shenk, Ray Uehara, Eckart Voland, Kai Willführ, Bruce Winterhalder, John Ziker, Chris von Rueden

*J. R. Soc. Interface* **15**, 20180035 (Published Online 18 July 2018) (doi:10.1098/rsif.2018.0035)

With apologies, an incorrect author list was given in the article. An additional author, Chris von Rueden, (Jepson School of Leadership Studies, University of Richmond) also contributed to this work. Author Ryan Schacht's affiliation was out-of-date. His correct affiliation is: Department of Anthropology, East Carolina University.

