# Supplementary material for: Greater wealth inequality, less polygyny: rethinking the polygyny threshold model
Source: J R Soc Interface. 2018 Jul 18;15(144):20180035. doi: 10.1098/rsif.2018.0035 (PMC6073648; doi:10.1098/rsif.2018.0035)
Supplement: Appendix [file rsif20180035supp1.pdf]

Supplementary Materials to: "Greater wealth inequality, less polygyny: Rethinking the polygyny threshold model"

Cody T. Ross,[1,2*] Monique Borgerhoff Mulder,[3*] Seung-Yun Oh,[4*] Samuel Bowles,[1*] Bret Beheim,[2] John Bunce,[2] Mark Caudell,[5] Gregory Clark,[3] Heidi Colleran,[6] Carmen Cortez,[3] Patricia Draper,[7] Russell D. Greaves,[8] Michael Gurven,[9] Thomas Headland,[10] Janet Headland,[10] Kim Hill,[11] Barry Hewlett,[12] Hillard S. Kaplan,[13] Jeremy Koster,[14] Karen Kramer,[8] Frank Marlowe,[15] Richard McElreath,[2] David Nolin,[16] Marsha Quinlan,[12] Robert Quinlan,[12] Caissa Revilla-Minaya,[17] Brooke Scelza,[18] Ryan Schacht,[8] Mary Shenk,[16] Ray Uehara,[10] Eckart Voland,[19] Kai Willführ,[21] Bruce Winterhalder,[3] John Ziker[20]

[1]Santa Fe Institute. NM. USA. [2]Max Planck Institute for Evolutionary Anthropology. Germany. [3]University of California, Davis. CA. USA. [4]Korea Insurance Research Institute, Republic of Korea. [5]Washington State University, Pullman. WA. USA. [6]Max Planck Institute for the Science of Human History. Germany. [7]University of Nebraska, Lincoln. NE. USA. [8]University of Utah. UT. USA. [9]University of California, Santa Barbara. CA. USA. [10]SIL International. TX. USA. [11]Arizona State University. AZ. USA. [12]Washington State University, Vancouver. WA. USA. [13]University of New Mexico. NM. USA. [14]University of Cincinnati. OH. USA. [15]University of Cambridge. UK. [16]Pennsylvania State University. PA. USA. [17]Vanderbilt University. TN. USA. [18]University of California, Los Angeles. CA. USA. [19]Institut für Philosophie der Universität Giessen. Germany [20]Boise State University. ID. USA. [21]Carl von Ossietzky University of Oldenburg. Germany.

Contents

1 Notes on the data taken from the Standard Cross-Cultural Sample 2

2 Wealth proxies 2

3 Percent female polygyny at equilibrium 3

4 An alternative definition of decreasing fitness returns to increasing number of wives 5

5 Visualizing model predictions as m_r and θ are varied 6

6 On the ratio of percent female polygyny to the rival wealth Gini coefficient 7

7 Estimating percent rich 7

8 Historical agricultural populations . 8

9 Estimating wealth elasticities 8

10 Age-adjustment 10

11 Model fit 11

12 Replication and open science 11

1 Notes on the data taken from the Standard Cross-Cultural Sample

In two cases, we needed to revise the Standard Polygamy Code (variable ♯861): for Toda, and for Basseri.

The Toda were coded as 0 ("polyandry," even though Cultural Basis for Polygamy (variable ♯860) is coded as 2 ("monogamy preferred but exceptional cases of polygyny"); we thus changed Standard Polygamy (variable ♯861) to 2 ("Monogamy preferred, but exceptional cases of polygyny") on basis of White [1], who refers to exceptional cases of polygyny.

The Basseri have a missing value for variable ♯861. From White [1] and Barth [2, page 107], we coded (conservatively) Standard Polygamy ♯861 as 3 ("Limited polygyny <20% of married males") on the basis of: "wealthy herd owners, with additional labor needs, frequently have plural wives who extend a man's fecundity in a way that saps his wealth." It could plausibly be recorded as 4 (>20%) [2].

2 Wealth proxies

A possible concern related to the cross-cultural compatibility of our estimates is that our rival wealth proxies vary between populations and productions systems—see Table 1 from the main text. As such, it remains possible that variation in the wealth measures used is responsible for variation in our estimates. For example, had we chosen a different rival wealth proxy for a given population—e.g., the value of household items instead of land owned—we may have obtained a different estimate of μ, and hence δ, in that population. In cross-cultural projects as wide-ranging as this one, however, there is rarely a single variable that can be compared directly across populations—instead, we have relied on ethnographic accounts to identify which sources of wealth are most relevant to production and reproduction in each society, and attempted to build a cross-culturally comparable data set by using the most locally relevant measures of wealth in each population.

Our proxy of rival wealth in foraging societies (weight) is especially problematic insofar as it undoubtedly also captures important elements of non-rival wealth, such as health or foraging skill; however, our choice here is constrained by the very fact that foragers hold small and relatively unvarying amounts of material wealth. We therefore have little choice but to treat weight as a proxy for access to material resources. Little in our overall argument, however, is affected by this methodological choice in foraging populations.

Among agriculturalists, the Polish sample is notable for its relatively low Gini coefficient on

rival wealth (specifically, land). Similiar estimates have been shown in other studies [3]. This study site is situated in an area with poor soils and long, hard winters, and the area was never particularly well-suited for large-scale agriculture for these reasons [4]. More than 80% of farming households in the sample own land that is classified in the lowest official grades [4]. Most importantly, the population has a long history of small-holder farming and a partible inheritance system [4]. In concert, these social norm reduced plot sizes over time since the late 1700s, and have likely contributed to the smaller-than-typical Gini coefficient on rival wealth.

3 Percent female polygyny at equilibrium

If a man marries n women, the non-rival wealth available to each wife is g and the rival wealth available to each wife is $\frac{m-cn}{n}$. Here, m refers to the total rival wealth of a male, and c refers to the cost of mating investment. In the Oh et al. [5] model, each wife produces offspring as a function of the wealth she has been provided by the male, adjusted for the importance—γ and μ—of each type of wealth to fitness. The fitness, w, of a male is then given by the effective number of wives acquired by the male multiplied by their average fitness:

$$w = \underbrace{n^{\delta}}_{\substack{\text{Effective}\\ \text{number}\\ \text{of wives}}} \cdot \underbrace{g^{\gamma}\left(\frac{m-nc}{n}\right)^{\mu}}_{\substack{\text{Average fitness}\\ \text{per effective wife}}} \tag{1}$$

The parameters γ and μ are constrained to the unit interval reflecting the assumption—strongly confirmed in our empirical estimates—that the marginal fitness effect of additional wealth of either type, while positive, is either constant or diminishing as wealth increases. Note that rival and non-rival wealth are modeled as complementary inputs using a Cobb-Douglas function. This assumption formalizes the idea that having high non-rival wealth (like farming skill) with limited material wealth (like land, seed, and farming tools) is not as relevant to fitness as having farming skill in the presence of substantial amounts of such material resources. In other words, the multiplicative nature of the fitness function means that the marginal fitness effect of each kind of wealth is greater as the amount of the other kind of wealth increases. The parameter δ—which is key to our proposed resolution of the polygyny paradox—controls the extent of diminishing returns to increasing number of wives for reasons unrelated to the need to share a male's rival wealth among wives; a value of one indicates no such sources of diminishing returns, and an increasing extent of diminishing returns—reducing what we term

the *effective number of wives* below the empirically observed number *n*—is indicated by values of δ falling farther below one.

If we consider only two classes of men, the rich and poor, with the rich males being indexed by r and the poor by p, then females can optimize their reproductive success by pairing with a (possibly married) rich male so long as the following condition is satisfied:

$$\underbrace{g_p^{\gamma}(m_p - c)^{\mu}}_{\text{Fitness of singleton wife of a poor man}} \leq \underbrace{g_r^{\gamma}(m_r - nc)^{\mu} n^{\delta-\mu-1}}_{\text{Fitness as one of n wives of a rich man}} \quad (2)$$

Eqs. 1 and 2 determine the fitness of males and females. Without loss of generality, we can define m_r, m_p, and c in terms of units of m_p, and likewise with g_r and g_p. Then, from Eqs. 1 and 2, one can use the Karush-Kuhn-Tucker conditions to derive (see [5]) an analytic expression for the equilibrium number of wives of the rich men, n^*,

$$n^* = \frac{m_r(1 - \frac{\mu}{\delta})}{c} \quad (3)$$

if male demand is limiting, or if c, the cost of bridewealth and mating investments, is such that the market clears. If female supply is limiting, and the market does not clear, then there is no simple closed form solution for n^*.

If we look at the of derivative of n^* in Eq. 3 with respect to δ,

$$\frac{\partial n^*}{\partial \delta} = \frac{m_r \mu}{\delta^2 c} \quad (4)$$

we find that it is always positive. Thus, increasing the extent to which female fitness is diminished by additional wives (i.e., decreasing δ), drives down male demand for additional wives.

Determining the effect of greater diminishing returns to additional wives (i.e., a lower value of δ) when female supply is limiting is more challenging. If female supply is limiting, the value of n^* implied by the polygyny threshold inequality in Eq. 1.2 in the main text has no closed form solution. To address this challenge we proceed as follows. Suppose that the polygyny threshold in Eq. 1.2 from the main text were satisfied by an equality:

$$g_p^{\gamma}(m_p - c)^{\mu} = g_r^{\gamma}(m_r - nc)^{\mu} n^{\delta-\mu-1} \quad (5)$$

Now, by differentiating the right-hand side of Eq. 5 with respect to δ, we have:

$$\frac{\partial \left(g_r^{\gamma}(m_r - nc)^{\mu} n^{\delta-\mu-1} \right)}{\partial \delta} = g_r^{\gamma} n^{(\delta-\mu-1)} (m_r - cn)^{\mu} \log(n) \quad (6)$$

The value $g_r^{\gamma} n^{(\delta-\mu-1)} (m_r - cn)^{\mu} \log(n)$ is positive so long as $n > 1$—which will always be true if the prospective bride is to marry as a co-wife—and $m_r > nc$—which must also

be true if the original equality were satisfied. Since this derivative is positive for all plausible model parameters, a decrease in δ will decrease the prospective female's fitness with the wealthy man below what it was under the higher δ value.

It is then apparent from Eq 6 that a lower δ would, holding other terms constant, decrease the supply of females to polygynous marriage. A man who was just barely rich enough so that an unpaired woman would choose to marry him as wife number $(n+1)$ under the initial δ, would, under the lower δ, be unable to secure the unpaired woman's partnership. An increase in the extent of diminishing returns to additional wives (lower δ) therefore reduces both male demand for, and female supply to, polygynous marriage.

Under the Oh et al. model [5], if at least some women marry monogamously, and if Eq. 3 holds, then there is a closed form solution for P at equilibrium, given by:

$$P = \underbrace{s}_{\text{Sex ratio}} \cdot \underbrace{\theta}_{\text{Fraction of rich males}} \cdot \underbrace{\underbrace{m_r}_{\text{Wealth ratio}} \cdot \underbrace{\frac{(1-\frac{\mu}{\delta})}{c}}_{\text{Conversion factor}}}_{n^*\text{, wives per rich man}} \qquad (7)$$

This follows from the fact that in a population with N_m males and N_f females, and a given n-polygyny level, n^*, there will be θN_m males who marry a total of $\theta N_m n^*$ females. Percent female polygyny is then:

$$P = \frac{\theta N_m n^*}{N_f} = s\theta n^* = s\theta m_r \frac{(1-\frac{\mu}{\delta})}{c} \qquad (8)$$

Note that Eqs 7 and 8 should be confined to the unit interval, but this condition is not apparent from the definition. Following the Oh et al. model [5], we assume here that the parameters are such that at least some women marry monogamously. If the parameters of the model are such that P is greater than 1, it would imply that all wives are predicted to have cowives. Also following the Oh et al. model [5], we allow n^* to be a real value for analytical tractability. In reality, n^* would normally be an integer value.

4 An alternative definition of decreasing fitness returns to increasing number of wives

In the main text, we model decreasing returns to increasing number of wives using a term, δ, directly on wife number. An alternative approach would be to assume that female's biological and material contributions to reproduction are affected by number of wives. There may be declining returns to female fitness which depend on number of wives; for example, sexually transmitted inflections that lead to infertility could cause diminished biological contributions

to fitness for all wives of a given male [6], if polygyny enhances infection rates [7, 8]. Using another version of the Oh et al [5] model, we can define a rich male's fitness as:

$$w = \underbrace{n}_{\substack{\text{Number}\\ \text{of wives}}} \cdot \underbrace{\left(\frac{l}{n^{\alpha}}\right)^{\lambda} g^{\gamma} \left(\frac{m_r - nc}{n}\right)^{\mu}}_{\substack{\text{Fitness}\\ \text{per wife}}} \tag{9}$$

where λ gives the importance of each female's biological contribution to offspring production, l, and α controls the extent to which additional wives diminish a given female's ability to reproduce. When $\alpha = 0$, this model reduces to the Oh et al [5] model, but when $\alpha > 0$, additional wives diminish the fitness of other wives for reasons other than dilution of rival wealth resources.

If we assume that male demand is limiting, then n^* can be written as:

$$n^* = \frac{m_r(1 - (\lambda\alpha + \mu))}{c(1 - \lambda\alpha)} \tag{10}$$

but there is no simple solution for the female supply condition. If we look at the of derivative of n^* in Eq. 10 with respect to α:

$$\frac{\partial n^*}{\partial \alpha} = -\frac{m_r \lambda \mu}{c(1 - \lambda\alpha)^2} \tag{11}$$

we find that it is always negative. Increasing the extent to which females' biological contributions to reproduction are diminished by additional wives, drives down male demand for additional wives. This model leads to the same qualitative findings as in the main analysis. Empirically, it is possible that polygyny might enhance female reproductive rates (e.g., $\alpha < 0$) [9], but evidence for such an effect in humans is very limited [10].

5 Visualizing model predictions as m_r and θ are varied

Here we present visualizations of the model predictions as m_r and θ are varied for various values of δ and μ. We hold the sex ratio constant at 1, and the cost of mating investment constant at 0.95. These figures demonstrate a few key facts: 1) polygyny is monotonically decreasing with increases in the population density of the poor, $1 - \theta$, 2) the rival wealth Gini is non-monotonic in the population density of the poor, 3) the ratio of percent polygyny to the wealth Gini is monotonically decreasing with increases in the population density of the poor, 4) both polygyny and wealth inequality are monotonically increasing with increases in m_r, and 5) polygyny is lower when the value $\delta - \mu$ is small.

[Figure 1 about here.]

6 On the ratio of percent female polygyny to the rival wealth Gini coefficient

In the main text, we state that the partial derivative of the Gini coefficient with respect to θ is negative whenever:

$$\theta > \frac{\sqrt{m_r} - 1}{m_r - 1} \tag{12}$$

To calculate this, we write:

$$\frac{\partial G(\theta, m_r)}{\partial \theta} = \frac{m_r}{(1 + (m_r - 1)\theta)^2} - 1 \tag{13}$$

and then elvauate when the right-hand side of Eq. 13 is less than 0.

From the main text, if male demand is limiting, then percent female polygyny is given by:

$$P = s\theta M_r(\delta - \mu)\frac{1}{\delta c} \tag{14}$$

The Gini coefficient on rival wealth is given by:

$$G = \frac{\theta m_r}{\theta m_r + (1 - \theta)} - \theta \tag{15}$$

The ratio of percent female polygyny to wealth inequality, P/G, is thus:

$$\frac{s\theta m_r(\delta - \mu)\frac{1}{\delta c}}{\frac{\theta m_r}{\theta m_r + (1-\theta)} - \theta} \tag{16}$$

so long as the parameters are such that $0 < P < 1$. The derivative of Eq. 16 with respect to θ is:

$$\frac{s m_r^2(\delta - \mu)}{c(m_r - 1)(\theta - 1)^2} \tag{17}$$

This value is positive as long as $\delta > \mu$, $m_r > 1$, and $s, c > 0$, which are fundamental assumptions of the model. As such, the ratio of percent female polygyny to the Gini coefficient on rival wealth is monotonic and decreasing as θ, the fraction of rich males, decreases towards 0. As wealth becomes concentrated by a small, rich elite, there will be lower levels of polygyny for a given level of wealth inequality.

7 Estimating percent rich

In our theoretical model, we assume a discrete two-class wealth distribution, but empirical wealth data typically have continuous distributions. To measure percent rich, we consider the rival wealth distribution, M, of a single population with N males, sorted in decreasing order, and define the condition:

$$\sum_{n=1}^{X} M_{[n]} \geq \phi \sum_{n=1}^{N} M_{[n]} \tag{18}$$

Then, for a given $\phi \in (0, 1)$, we can calculate the minimum number of men in the population, X, needed to satisfy the inequality. We can then define the frequency of rich men, θ, as the frequency of men in the upper ϕ percentile of cu-

mulative wealth in the population:

$$\theta = \frac{X}{N} \tag{19}$$

For our main analysis, we set $\phi = \{0.33, 0.50, 0.66\}$, yielding a measure of the minimal percentage of men that own one third, one half, or two thirds of the wealth in the population. This mathematical formulation is similar to identifying the point on a Lorenz curve that intersects a horizontal line at ϕ, and then dropping a vertical line down from this intersection point to the x-axis. We can then calculate 1 minus the distance between the intersection point of the vertical line with the x-axis and the origin to yield θ.

In the final, empirically motivated estimate—ψ in the main text—we calculate the average wealth of men with one and two wives, and define ψ to be the percentage of men who have more wealth than the average of these two numbers.

8 Historical agricultural populations

To identify additional agricultural populations with which we could test for a decreasing frequency of rich males relative to other subsistence modes, we searched in Fochesato and Bowles [11, Table 15]. This table compiled papers mentioning Gini coefficients on wealth in historical populations. We searched each table entry until the 1500s. Open access papers were scanned for Lorenz plots, or data that could be used to generate them. We then used the Lorenz curves to graphically estimate the minimum fraction of males that possess the upper ϕ percent of cumulative wealth in each population. The populations added in this supplementary analysis are listed in Table 1. Our analysis is robust to inclusion or exclusion of the two populations where income rather than wealth estimates were provided.

[Table 1 about here.]

We replicate Fig. 6 from the main text in Fig. 2. We find that our results in the this supplementary analysis are qualitatively similar to our findings in the main analysis, but our confidence intervals are now much narrower in support of our predictions.

[Figure 2 about here.]

9 Estimating wealth elasticities

To estimate the importance of rival wealth and wives to reproductive success in each population, p, we use a Cobb-Douglas function, which implies that the log of predicted reproductive

success in male i, $\Lambda_{[i]}$, is given by:

$$\log(\Lambda_{[i]}) = \beta_{[p(i),1]} + \beta_{[p(i),2]} \log(E_{[i]}) + \beta_{[p(i),3]} \log(W_{[i]} + \eta_{[i]}) + \beta_{[p(i),4]} \log(M_{[i]}) \quad (20)$$

where each male's exposure time to risk of reproductive success (i.e., number of years lived in the age range between 13 and 60 years) is given by $E_{[i]}$, number of marriages is given by $W_{[i]}$, and rival wealth is given by $M_{[i]}$. The vector β gives the unknown parameters unique to the population, p, of individual i. Note that the parameter $\eta_{[i]}$ is equal to 0 if $W_{[i]} > 0$, and is defined as $\hat{\eta}_{[p(i)]} \in (0, 1)$—a parameter to be estimated—otherwise. This method allows men with no wives to produce offspring. Note that $\hat{\eta}_{[p]}$ represents the effective exposure to mating chances outside of marriages in population p, and is constrained by fiat to be less than the mating chances inside a marriage. Eq. (20) is thus an empirically estimable approximation to the male reproductive success function described in the theoretical model of the main paper.

The function for reproductive success, $R_{[i]}$, is then defined using a Negative Binomial outcome distribution:

$$R_{[i]} \sim \text{Negative Binomial}(\Lambda_{[i]} B_{[p(i)]}, B_{[p(i)]}) \quad (21)$$

where the term $\Lambda_{[i]} B_{[p(i)]}$ defines the shape parameter of a Gamma distribution, and $B_{[p(i)]}$ defines the inverse scale parameter.

Our measurement of reproductive success is motivated by historical evidence that number of children born predicts number of grandchildren [12], making the former a proxy measure of fitness. Our proxies for reproductive success vary somewhat across populations; for example, some data contributors report number of living children, some report number of children surviving to at least age five, while others report children surviving to reproductive age. Typically, survival to age 5 is used in comparative studies of reproductive success in the developing world [13], because most mortality occurs prior to the fifth birthday; mortality after the fifth birthday is generally low. While best estimates of fitness are drawn from multigenerational data [12, 14], studies of contemporary demography are limited by their very nature as contemporary and must rely on slightly noisier proxy measures of fitness.

Rival wealth holdings are estimated using one or two proxy measures, $\hat{M}_{[i,1]}$ and $\hat{M}_{[i,2]}$. If we have only a single proxy measure, then: $M_{[i]} = \hat{M}_{[i,1]}$. To convert two wealth proxies into a single kind of rival currency, we integrate endogenously estimated shadow prices, ς, into

Eq. (20):

$$M_{[i]} = \hat{M}_{[i,1]} + \varsigma_{[p(i)]}\hat{M}_{[i,2]} \quad (22)$$

In cases where the vectors containing $\hat{M}_{[i,1]}$ or $\hat{M}_{[i,2]}$ contain zeros, we add a small constant. In the case of foragers, where weight was used as a proxy for wealth, we subtract a constant slightly less than the minimum weight value in order to yield a reasonable location for the zero of the wealth vector.

The shadow price parameters are given log-normal priors:

$$\varsigma_{[p]} \sim \text{Log Normal}(0, 1) \quad (23)$$

The parameters controlling reproduction outside of marriages are given uniform priors on the unit interval:

$$\hat{\eta}_{[p]} \sim \text{Beta}(1, 1) \quad (24)$$

The inverse scale parameters are given weak but proper, positive-constrained priors:

$$B_{[p]} \sim \text{Cauchy}(0, 5)T[0,] \quad (25)$$

where the symbol $T[0,]$ indicates truncated support > 0.

We use a multi-level model to estimate the elasticity parameters:

$$\beta_{[p]} \sim \text{Multivariate Normal}(\Theta, \Omega) \quad (26)$$

The elements of Θ are given weak priors:

$$\Theta \sim \text{Normal}(0, 5) \quad (27)$$

The covariance matrix, Ω, is defined as:

$$\Omega = \text{Diag}(\sigma)\rho\text{Diag}(\sigma) \quad (28)$$

where the elements of σ have weak but proper, positive-constrained priors:

$$\sigma \sim \text{Cauchy}(0, 5)T[0,] \quad (29)$$

10 Age-adjustment

In order to use all cohorts of the adult male population, relevant measures—wives and wealth—are age-adjusted to represent their predicted values at the age of 60 years. To age-adjust wealth, we model:

$$\hat{M}_{[i,1]} \sim \text{Gamma}(\exp(\alpha_{[p(i),1]} + \alpha_{[p(i),2]} \log(E_{[i]}))Z_{[p(i)]}, Z_{[p(i)]}) \quad (30)$$

An estimate of completed wealth, $\bar{M}_{[i,1]}$ is then:

$$\bar{M}_{[i,1]} = \hat{M}_{[i,1]} + Q_{[i,1]} \quad (31)$$

where:

$$Q_{[i,1]} \sim \text{Gamma}\big(e^{\alpha_{[p(i),1]}}\big(\bar{E}^{\alpha_{[p(i),2]}} - E_{[i]}^{\alpha_{[p(i),2]}}\big)Z_{[p(i)]}, Z_{[p(i)]}\big) \quad (32)$$

Note that $\bar{E} = 60$ gives the maximum possible exposure time to risk of acquisition. As such, the first term in Eq. 31, $\hat{M}_{[i,1]}$, gives how much wealth has been acquired by exposure time $E_{[i]}$, and the second term, $Q_{[i,1]}$, gives an estimate of the additional wealth that will be acquired between times $E_{[i]}$ and $\bar{E}$. This model adjusts the estimates of a wealth proxy for exposure-time associated structuring of both mean and variance. Further, this adjustment method propagates uncertainty in the predicted quantity of wealth that will be obtained by younger men. We use the same model structure for the other wealth proxy and wives. The completed wealth proxies are combined using the estimated shadow prices. The percent rich and percent female polygyny measures presented in the main paper are based on the completed wealth and wife estimates. See model code for additional details.

11 Model fit

Model fit was checked using Stan's $\hat{r}$ and effective sample size measures. Additionally, we checked traceplots from a sample of model parameters, finding both chains to have settled in the same posterior region. Both chains mixed well. See Fig. 3.

[Figure 3 about here.]

12 Replication and open science

Interested readers can replicate our analysis using the model code and data included at: https://github.com/ctross/publications/polygynypuzzle. Analysis is conducted using R [15] and rstan [16].

1. White, D. R. *et al.* Rethinking polygyny: co-wives, codes, and cultural systems [and comments and reply]. *Current Anthropology* **29**, 529–572 (1988).

2. Barth, F. *Nomads of south Persia—The Basseri tribe of the Khamseh confederacy* (Read Books Ltd, 2013 [1961]).

3. Colleran, H., Jasienska, G., Nenko, I., Galbarczyk, A. & Mace, R. Fertility decline and the changing dynamics of wealth, status and inequality. *Proceedings of the Royal Society of London B: Biological Sciences* **282** (2015). URL http://rspb.royalsocietypublishing.org/content/282/1806/20150287.

4. Colleran, H. Farming in transition: land and property inheritance in a rural Polish population. *Soc. Biol. Hum. Aff* **78**, 7–19 (2014).

5. Oh, S. Y., Ross, C., Borgerhoff Mulder, M. & Bowles, S. The decline of polygyny: An interpretation. *Santa Fe Institute Working Paper* (2017). URL https://sfi-edu.s3.amazonaws.com/sfi-edu/production/uploads/working_paper/pdf/2017-12-037_ae5724.pdf.

6. Bauch, C. & McElreath, R. Disease dynamics and costly punishment can foster socially imposed monogamy. *Nature Communications* **7**, 1–9 (2016). URL http://www.nature.com/ncomms/2016/160412/ncomms11219/full/ncomms11219.html.

7. Mah, T. L. & Halperin, D. T. Concurrent sexual partnerships and the HIV epidemics in Africa: Evidence to move forward. *AIDS and Behavior* **14**, 11–16 (2010). URL https://doi.org/10.1007/s10461-008-9433-x.

8. Reniers, G. & Watkins, S. Polygyny and the spread of HIV in Sub Saharan Africa: a case of benign concurrency. *AIDS (London, England)* **24**, 299 (2010).

9. Altmann, S. A., Wagner, S. S. & Lenington, S. Two models for the evolution of polygyny. *Behavioral Ecology and Sociobiology* **2**, 397–410 (1977). URL https://doi.org/10.1007/BF00299508.

10. Chisholm, J. S. & Burbank, V. K. Monogamy and polygyny in southeast arnhem land: male coercion and female choice. *Ethology and Sociobiology* **12**, 291–313 (1991).

11. Fochesato, M. & Bowles, S. Wealth inequalities over the past nine thousand years. *Santa Fe Institute Working Paper* (2018).

12. Bolund, E. & Lummaa, V. The effects of resource availability and the demographic transition on the genetic correlation between number of children and grandchildren in humans. *Heredity* **118**, 186–192 (2017).

13. Borgerhoff Mulder, M. *et al.* Intergenerational wealth transmission and the dynamics of inequality in small-scale societies. *Science* **326**, 682–688 (2009).

14. Goodman, A., Koupil, I. & Lawson, D. W. Low fertility increases descendant socioeconomic position but reduces long-term fitness in a modern post-industrial society. *Proceedings of the Royal Society of London B: Biological Sciences* **279**, 4342–4351 (2012).

15. R Core Team. *R: A Language and Environment for Statistical Computing*. R Foundation for Statistical Computing, Vienna, Austria (2016). URL https://www.R-project.org/.

16. Stan Development Team. RStan: the R interface to Stan (2016). URL http://mc-stan.org/. R package version 2.14.1.

17. Windler, A., Thiele, R. & Muller, J. Increasing inequality in Chalcolithic Southeast Europe: the case of Durankulak. *Journal of Archaeological Science* **40**, 204–210 (2013).

18. Kron, G. The distribution of wealth at Athens in comparative perspective. *Zeitschrift für Papyrologie und Epigraphik* 129–138 (2011).

19. Bagnall, R. S. Landholding in late Roman Egypt: the distribution of wealth. *Journal of Roman Studies* **82**, 128–149 (1992).

20. Scheidel, W. & Friesen, S. J. The size of the economy and the distribution of income in the Roman Empire. *The Journal of Roman Studies* **99**, 61–91 (2009).

21. Milanovic, B. An estimate of average income and inequality in Byzantium around year 1000. *Review of Income and Wealth* **52**, 449–470 (2006).

22. Blanshei, S. R. Population, wealth, and patronage in medieval and renaissance Perugia. *The Journal of Interdisciplinary History* **9**, 597–619 (1979).

23. Smith, M. E., Dennehy, T., Kamp-Whittaker, A., Colon, E. & Harkness, R. Quantitative measures of wealth inequality in ancient central Mexican communities. *Advances in Archaeological Practice* **2**, 311–323 (2014).

List of Figures

1 Visualization of model predictions. We assume that male demand is limiting and that $s = 1$ and $c = 0.95$. We vary (1-θ) from 0 to 1. The colors represent m_r values of: 2.5, 3.5, 5.5, 9.5, 17.5, and 33.5, as black shifts to purple. Note that values in the last frame are only displayed for parameter combinations in which $0 < P < 1$. 15

2 Frequency of rich males. Frames (a), (b), and (c) illustrate the minimal fraction of men who possess the upper ϕ percent of cumulative wealth in the population. We see that wealth in agricultural populations is disproportionately possessed by a significantly smaller fraction of the population than in horticultural or even agropastoral societies. Values plotted in the legends show the mean difference (and 90% confidence intervals) in the frequency of rich males between the focal subsistence type and agricultural populations. For example, in frame (a) the estimate of the mean frequency of rich males in horticultural populations was 0.10 (90%CI: 0.08, 0.13) higher than the corresponding mean estimate in agricultural populations. . . . 16

3 Traceplots from a random sample of model parameters. 17

Figure 1: Visualization of model predictions. We assume that male demand is limiting and that $s = 1$ and $c = 0.95$. We vary (1-θ) from 0 to 1. The colors represent m_r values of: 2.5, 3.5, 5.5, 9.5, 17.5, and 33.5, as black shifts to purple. Note that values in the last frame are only displayed for parameter combinations in which $0 < P < 1$.

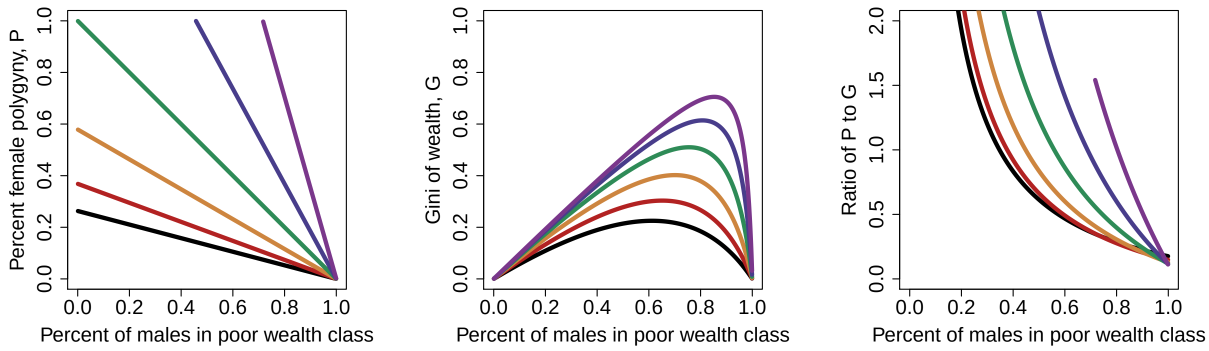

(a) $\delta = 0.5$ and $\mu = 0.45$.

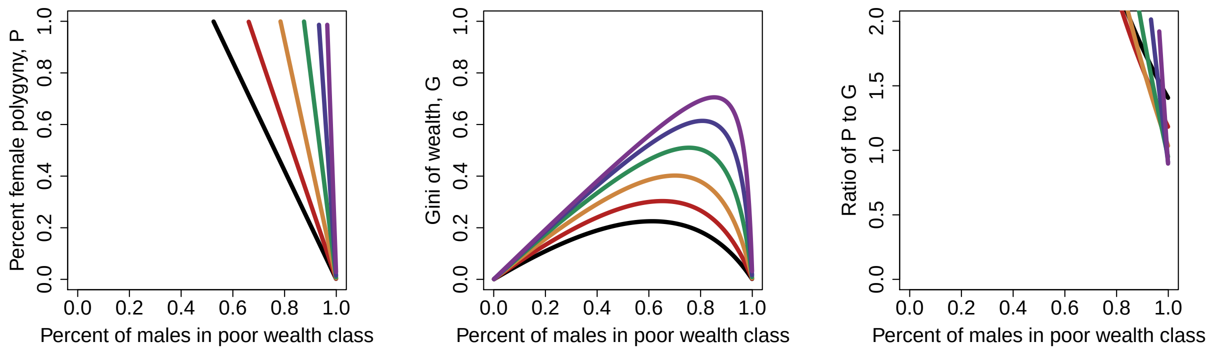

(b) $\delta = 0.5$ and $\mu = 0.10$.

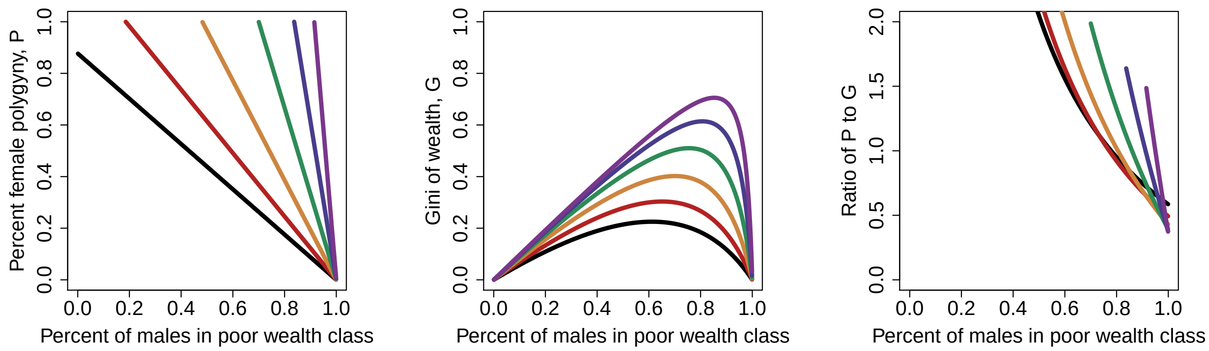

(c) $\delta = 0.15$ and $\mu = 0.10$.

Figure 2: Frequency of rich males. Frames (a), (b), and (c) illustrate the minimal fraction of men who possess the upper ϕ percent of cumulative wealth in the population. We see that wealth in agricultural populations is disproportionately possessed by a significantly smaller fraction of the population than in horticultural or even agropastoral societies. Values plotted in the legends show the mean difference (and 90% confidence intervals) in the frequency of rich males between the focal subsistence type and agricultural populations. For example, in frame (a) the estimate of the mean frequency of rich males in horticultural populations was 0.10 (90%CI: 0.08, 0.13) higher than the corresponding mean estimate in agricultural populations.

(a) Pct. of men with ≥ 0.33 of total wealth

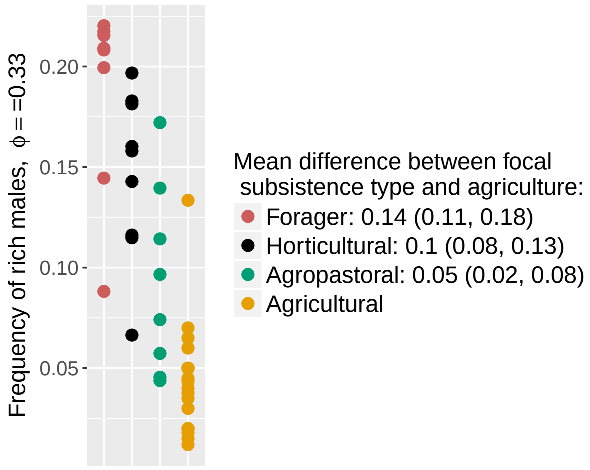

(b) Pct. of men with ≥ 0.50 of total wealth

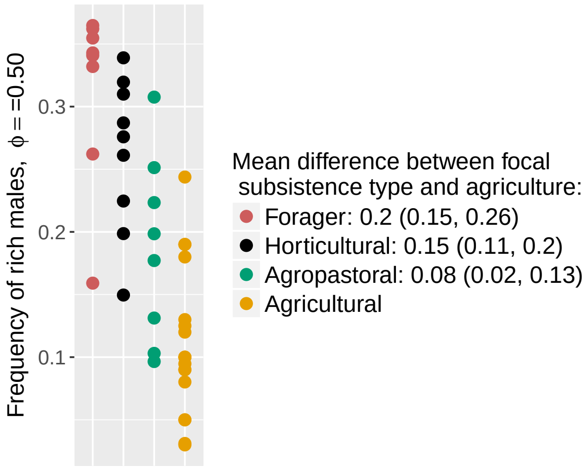

(c) Pct. of men with ≥ 0.66 of total wealth

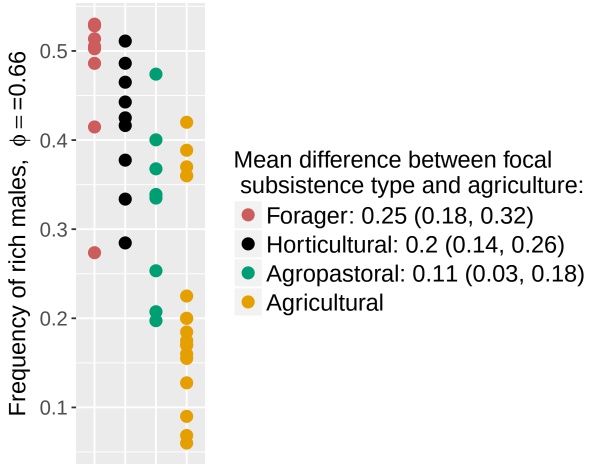

Figure 3: Traceplots from a random sample of model parameters.

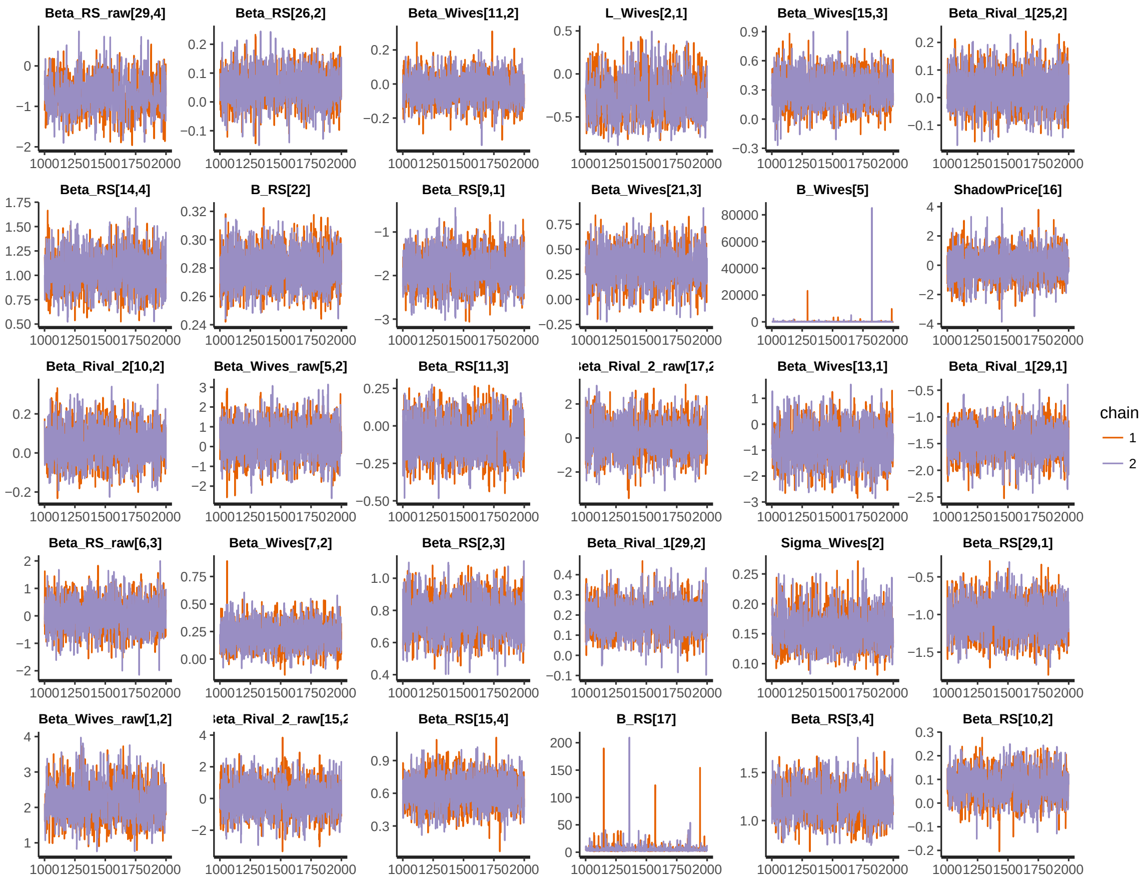

List of Tables

1 A sample of historical agricultural and/or material wealth limited populations. The numerical columns reflect the value of the frequency of rich males, θ, when the value of ϕ, the cumulative wealth owned by the rich class, is set at various values. . 19

Table 1: A sample of historical agricultural and/or material wealth limited populations. The numerical columns reflect the value of the frequency of rich males, θ, when the value of ϕ, the cumulative wealth owned by the rich class, is set at various values.

Year	Site	$\theta, \phi = 0.33$	$\theta, \phi = 0.50$	$\theta, \phi = 0.66$	Source	Figure	Proxy
5100 BCE	Hamangia I and II	0.04	0.10	0.16	[17]	Figure 4	Value of grave goods
4800 BCE	Hamangia III	0.04	0.10	0.20	[17]	Figure 4	Value of grave goods
4550 BCE	Hamangia IV	0.05	0.10	0.18	[17]	Figure 4	Value of grave goods
4350 BCE	Varna I	0.07	0.13	0.23	[17]	Figure 4	Value of grave goods
4350 BCE	Varna II and III	0.02	0.05	0.09	[17]	Figure 4	Value of grave goods
321 BCE	Athens	0.02	0.05	0.16	[18]	Figure 2	Estimated wealth
116 BCE	Hermopolite	0.02	0.03	0.06	[19]	Figure 1	Land
150	Roman Empire	0.03	0.13	0.37	[20]	Figure 3	Income
1000	Byzantium	0.02	0.19	0.42	[21]	Table 7	Income
1258	Italy	0.05	0.09	0.17	[22]	Table 1	Wealth
1498	Italy	0.05	0.09	0.17	[22]	Table 1	Wealth
1511	Italy	0.07	0.12	0.20	[22]	Table 1	Wealth
1540	Quauhchichinollan	0.06	0.19	0.36	[23]	Supp. Data	Land
1540	Huitzillan	0.05	0.18	0.36	[23]	Supp. Data	Land
